# Chemical Characterization and Metabolic Profiling of the Compounds in the Chinese Herbal Formula Li Chang Decoction by UPLC-QTOF/MS

**DOI:** 10.1155/2022/1322751

**Published:** 2022-04-13

**Authors:** Baofu Lin, Shaoju Guo, Xinxin Hong, Xiaoyan Jiang, Haiwen Li, Jingwei Li, Linglong Guo, Mianli Li, Jianping Chen, Bin Huang, Yifei Xu

**Affiliations:** Shenzhen Traditional Chinese Medicine Hospital, The Fourth Clinical Medical College of Guangzhou University of Chinese Medicine, Shenzhen 518033, China

## Abstract

**Background:**

Li Chang decoction (LCD), a Chinese medicine formula, is commonly used to treat ulcerative colitis (UC) in clinics.

**Purpose:**

This study aimed to identify the major components in LCD and its prototype and metabolic components in rat biological samples.

**Methods:**

The chemical constituents in LCD were identified by establishing a reliable ultra-performance liquid chromatography coupled with quadrupole time-of-flight tandem mass spectrometry (UPLC-QTOF/MS) method. Afterwards, the rats were orally administered with LCD, and the biological samples (plasma, urine, and feces) were collected for further analyzing the effective compounds in the treatment of UC.

**Result:**

A total of 104 compounds were discriminated in LCD, including 26 flavonoids, 20 organic acids, 20 saponins, 8 amino acids, 5 oligosaccharides, 5 tannins, 3 lignans, 2 alkaloids, and 15 others (nucleosides, glycosides, esters, etc.). About 50 prototype and 94 metabolic components of LCD were identified in biological samples. In total, 29 prototype components and 22 metabolic types were detected in plasma. About 27 prototypes and 96 metabolites were discriminated in urine, and 34 prototypes and 18 metabolites were identified in feces.

**Conclusion:**

The flavonoids, organic acids, and saponins were the major compounds of LCD, and this study promotes the further pharmacokinetic and pharmacological evaluation of LCD.

## 1. Introduction

Traditional Chinese medicine (TCM) attracts more attention in the world since it possesses reliable therapeutic efficacy in some complex diseases, especially chronic illness [1]. The chemical composition of Chinese herbal compound is complex, and the composition of the multi-Chinese medicine is crossed, summarized as “multitarget and multicomponent,” which is the feature of TCM [2, 3]. This characteristic promotes the curative effect and reduces toxicity; however, it brings enormous challenge to figure out the effective components and mechanism for the therapeutic effect [4].

**Figure 1:**
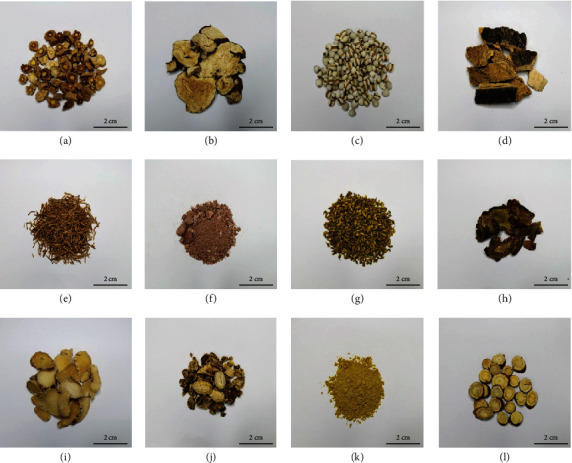
Decoction samples of 12 Chinese herbal medicines in LCD. (a) Codonopsis Radix; (b) Atractylodis Macrocephalae Rhizoma; (c) Coicis Semen; (d) Ailanthi Cortex; (e) Cynanchi Paniculati Radix et Rhizoma; (f) Halloysitum Rubrum; (g) Sophorae Flos; (h) Notoginseng Radix et Rhizoma; (i) Bletillae Rhizoma; (j) Chebulae Fructus; (k) Typhae Pollen; (l) Glycyrrhizae Radix et Rhizoma.

Li Chang decoction (LCD), a Chinese compound prepared from twelve Chinese medicine including Codonopsis Radix (CR), Notoginseng Radix et Rhizoma (NRR), Bletillae Rhizoma (BR), Sophorae Flos (SF), Glycyrrhizae Radix et Rhizoma (GRR), Cynanchi Paniculati Radix et Rhizoma (CPRR), Typhae Pollen (TP), Chebulae Fructus (CF), Atractylodis Macrocephalae Rhizoma (AMR), Ailanthi Cortex (AC), Coicis Semen (CS), and Halloysitum Rubrum (HR), has been commonly used to treat ulcerative colitis (UC) in clinics for over 20 years(Figure 1). UC is a chronic disease of inflammatory bowel diseases, which seriously impact the life quality of patients, and is sometimes life-threatening. LCD remarkably reduces the symptoms and recurrence rate of UC in clinical [5]. Although some of the major ingredients such as the polysaccharides from CR and AMR and rutin from SF have been proved effective in the treatment of UC, the effective components of LCD are still controversial and unclear [6–8]. Therefore, the systematic research on the effective component and metabolite profiling of LCD is an urgent need.

Ultra-performance liquid chromatography coupled with quadrupole time-of-flight tandem mass spectrometry (UPLC-QTOF/MS) provides a rapid and reliable method to identify the component of natural medicine, which promotes the development of natural medicine component analysis and new drug discovery [9, 10]. Herein, we recruited an UPLC-QTOF/MS method to profile the effective components of LCD, and the unknown components were classified and assigned based on the fragmentation patterns and diagnostic ions of different structural types of components. According to the component characterization result of LCD *in vitro*, the prototypes in plasma, urine, and feces were further analyzed based on the similarity of mass spectrometry behavior (accurate molecular weight and secondary fragments) and chromatographic behavior (retention time). Metabolites were matched *e* mass defect filtering (MDF) caused by biotransformation and were further confirmed by MS/MS spectrum analysis.

## 2. Material and Methods

### 2.1. Chemicals and Drugs

LCD was prepared by the Pharmaceutical Department, Shenzhen Traditional Chinese Medicine Hospital. The Chinese medicine including Codonopsis Radix (Lot: 190505101, root of *Codonopsis pilosula* (Franch.) Nannf.), Atractylodis Macrocephalae Rhizoma (Lot: 1904001, rhizoma of *Atractylodes macrocephala* Koidz.), Chebulae Fructus (Lot: 181203361, fructus of *Terminalia chebula* Retz.), Halloysitum Rubrum (Lot: 190300991), Sophorae Flos (Lot: 190504381, flos of *Sophora japonica* L.), Typhae Pollen (Lot: 190401, pollen of *Typha angustifolia* L.), Ailanthi Cortex (Lot: 181001, cortex of *Ailanthus altissima* (Mill.) Swingle), Bletillae Rhizoma (Lot:HX19K01, rhizoma of *Bletilla striata* (Thunb.) Reichb. f), Coicis Semen (Lot: 1905002, semen of *Coix lacryma-jobi* L. var. *ma-yuen* (roman) Stapf), Notoginseng Radix et Rhizoma (Lot: 190401411, radix and rhizoma of *Panax notoginseng* (Burk.) F. H. Chen), Cynanchi Paniculati Radix et Rhizoma (Lot: 190403711, radix and rhizoma of *Cynanchum paniculatum* (Bge.) Kitag.), and Glycyrrhizae Radix et Rhizoma (Lot: 1905001, radix and rhizoma of *Glycyrrhiza uralensis* Fisch.) was purchased from Kangmei Pharmaceutical Co., Ltd (Puning, China). Trigonelline, chebulic acid, gallic acid, 6,7-dihydroxycoumarin, corilagin, typhaneoside, rutin, hyperoside, liquiritin, nicotiflorin, lobetyolin, ginsenoside Re, ginsenoside Rg1, quercetin, ginsenoside Rb1, naringenin, 20S-ginsenoside Rh1, isorhamnetin, ginsenoside Rd, and glycyrrhizic acid, a total of 20 reference standards, were purchased from Chengdu Alfa Biotechnology Co., Ltd. The purity of each compound was more than 98% determined by the HPLC analysis. Methanol was of HPLC grade. Ultrapure water was obtained by the filtration of distilled water using a Milli-Q system (Millipore, USA). LC-MS grade acetonitrile was purchased from Fisher Scientific (Fair Lawn, New Jersey, USA), and LC-MS grade formic acid was purchased from Sigma-Aldrich (St, Missouri, USA).

### 2.2. Animal

Male Sprague-Dawley rats (300 ± 20 g) were obtained from the Medical Experimental Animal Center of Guangzhou University of Chinese Medicine, China. Rats were housed in specified pathogen-free conditions (23 ± 2°C) under a 12-h light/12-h dark cycle and given free access to food and water. The protocols were approved by the Animal Experimental Ethics Committee of Guangzhou University of Chinese Medicine (Guangzhou, China).

### 2.3. LCD Preparation

The Medicine Codonopsis Radix, Atractylodis Macrocephalae Rhizoma, Chebulae Fructus, Halloysitum Rubrum, Sophorae Flos, Typhae Pollen, Ailanthi Cortex, Bletillae Rhizoma, Coicis Semen, Notoginseng Radix et Rhizoma, Cynanchi Paniculati Radix et Rhizoma, and Glycyrrhizae Radix et Rhizoma were weighed and mixed at a ratio of 6 : 3:3 : 6:3 : 3:6 : 3:6 : 2:6 : 2. The total weight of LCD is 245g, and the mixture was extracted twice by boiling in distilled water, and eight times distilled water (1960 ml) (w/v) was used to boil for 40 min in the first time, which changes to four times distilled water (980 ml) (w/v) in the second time. The two extracts were merged and centrifuged at 3,000 rpm, for 5 min to exclude dregs, and the supernatant was concentrated to 3.185 g/ml under reduced pressure at 55°C.

### 2.4. Rat Treatment and Sample Collection

The dose of LCD used in this experiment is 22.05 g/kg, which is the biological equivalent dose of humans. Three rats were fasted for 12 h with free access to drinking water, and then, the rats were orally administered with LCD. LCD was diluted to 2.205 g/ml with distilled water before giving to rat. Then, the blood samples were collected in the heparin anticoagulant tube through retro-orbital plexus at 0.25, 0.5, 1, 2, 4, 6, 8, 10, and 12 h. The plasma samples were obtained by centrifugation at 3000 rpm for 10 min. Samples of the same point were combined and stored at −80°C until use. Feces and urine samples were collected during 0–12 h.

### 2.5. Biological Sample Preparation

For the plasma sample, about 200 *μ*l plasma was mixed with 600 *μ*l acetonitrile (containing 0.2% methanoic acid). After vortexing for 2 min, the samples were centrifuged at 13000 rpm, 4°C, 10 min. Then, 400 *μ*l supernatant was removed, dried under nitrogen gas, and redissolved in 100 *μ*l acetonitrile (50%). Finally, the samples were centrifuged at 13000 rpm, 4°C, 10 min, and a 2 *μ*l aliquot was injected into UPLC-QTOF-MS.

For the fecal sample, about 300 mg of feces was weighed and mixed with 1 ml methanol. After the addition of magnetic beads, the samples were homogenized using tissue grinders (Shanghai Jingxin, Shanghai, China) and centrifuged at 13000 rpm, 4°C, 10 min. About 200 *μ*l supernatant was removed, dried under nitrogen gas, and redissolved in 200 *μ*l acetonitrile (50%). Finally, the samples were centrifuged at 13000 rpm, 4°C, 10 min, and a 2 *μ*l aliquot was injected into UPLC-QTOF-MS.

For the urine sample, the mixed urine was centrifuged at 4000 rpm for 10 min, and 1 ml supernatant was loaded on pre-activated Sep-Pak Vac C18 columns (3 cc, 500 mg, Waters, Ireland). After washing with 1 ml ultrapure water and eluting with 1 ml methanol, the elution was collected and centrifuged at 13000 rpm, 4°C, 10 min. About 400 *μ*l supernatant was transferred and dried under nitrogen gas. The residues were redissolved in 400 *μ*l acetonitrile (50%). Finally, the samples were centrifuged at 13000 rpm, 4°C, 10 min, and a 2 *μ*l aliquot was injected into UPLC-QTOF-MS.

### 2.6. UPLC-QTOF-MS Analysis Condition

The separation equipment for this assay was Sciex Exion LC, and the chromatographic column was Waters Acquity HSS T3 (2.1 × 150 mm, 1.7 *μ*m). The temperature was set at 35°C, and the flow rate was 0.3 ml/min. The mobile phases were 0.1% formic acid in water (A) and acetonitrile (B), with the optimized gradient as follows: 0–5 min from 3% B to 8% B, 5–11 min from 8% B to 30% B, 11–20 min from 30% B to 80% B, 20–21 min from 80% B to 95% B, 21–25 min was maintained at 95% B, and then back to the initial ratio and re-equilibration for 7 min.

The 5600 QTOF mass spectrometer (AB Sciex, Foster City, CA, USA) equipped with an ESI ion source was operated in positive and negative modes, and the mass range was *m/z* of 100–1250. The details of mass spectrometry conditions were summarized as follows: gas 1 and gas 2, 45 psi; curtain gas, 35 psi; heat block temperature, 500°C; ion spray voltage, −4.5 kV in negative mode and 5.5 kV in positive; declustering potential, 50V; collision energy, ±35 V; and the collision energy spread (CES), ±15 V. Sciex OS 1.6.1 was the basal data processing platform, and MetabolitePilot 2.0.4 software was applied for further metabolite fishing.

## 3. Results and Discussion

### 3.1. Characterization of Chemical Compounds in LCD

**Figure 2:**
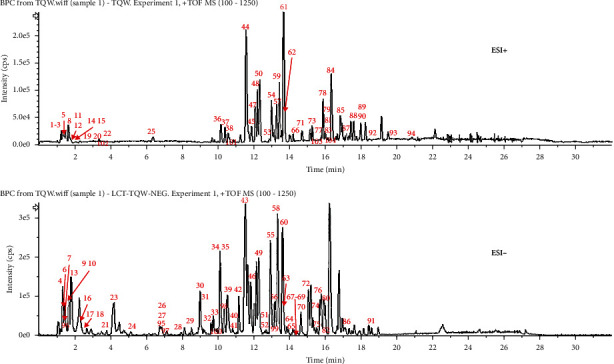
Base peak chromatogram (BPC) of LCD.

The base peak chromatograms of LCD in negative and positive ion modes are shown in Figure 2. A total of 104 chemical components, including 20 saponins, 26 flavonoids, 5 tannins, 20 organic acids, 8 amino acids, 2 alkaloids, 5 oligosaccharides, and 3 lignans, were identified or tentatively characterized by UPLC-QTOF-MS. As the result of chemical composition classification is summarized in Table 1, CR mainly contained alkaloid compounds and oligosaccharides, while NRR was characterized by saponins. Besides, the major constituents of SF were flavonoids. GRR contains saponins and flavonoids, and CPRR was as characterized by the C21 type steroidal saponins. The characteristic ingredients of TP were flavonoids and organic acids. CF was characterized by the component of tannins; AMR contains organic acids and esters.

**Figure 3:**
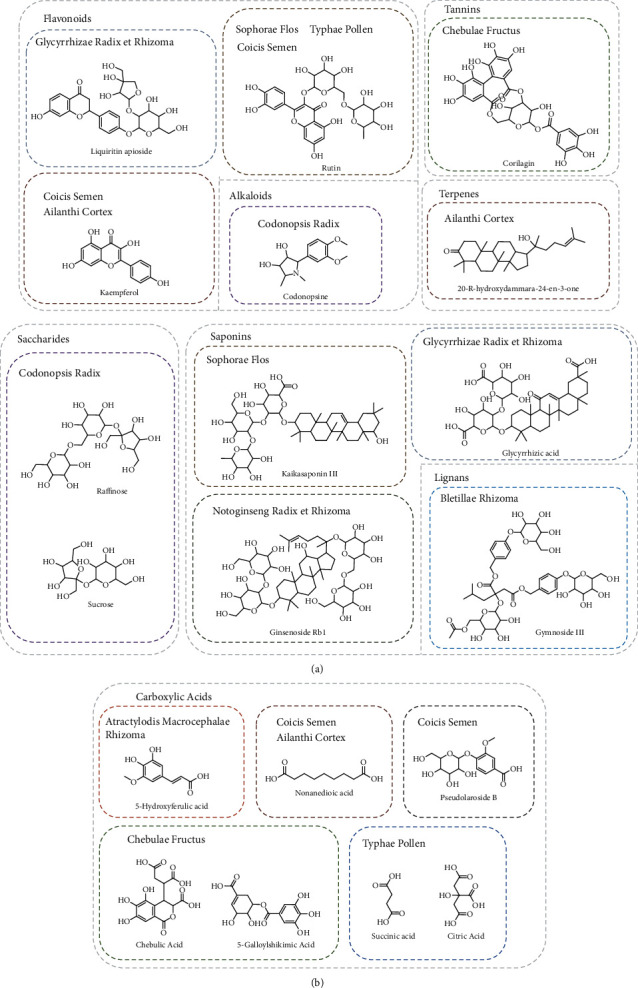
Representative structures of each medicine of LCD.

Generally, the characteristic components of AC were triterpenes, and the CS was characterized by lipids. However, both chemical categories were difficult to extract by water so that only flavonoids and organic acids in AC and CS were still detected and identified.  Figure 3 draws the part of representative structures of each medicine.

### 3.2. Fragmentation Mechanisms of Medicine Representative Structures

#### 3.2.1. Codonopsis Radix-Derived Compounds

**Figure 4:**
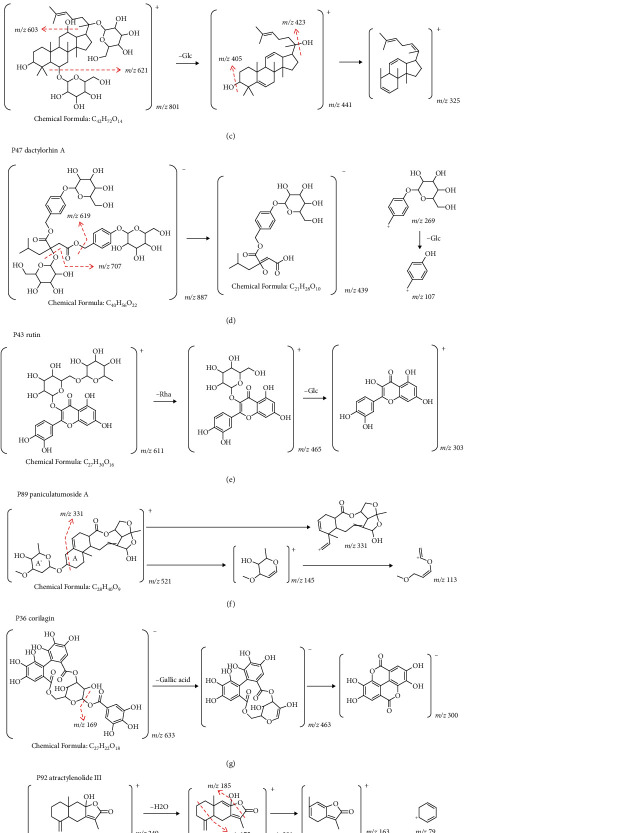
MS/MS spectrum and major fragmentation pathways of representative structure in LCD. (a) P14 verbascose; (b) P5 trigonelline; (c) P59 ginsenoside Rg1; (d) P47 dactylorhin A; (e) P43 rutin; (f) P89 paniculatumoside A; (g) P36 corilagin; (h) P92 atractylenolide III; (i) P104 20-R-hydroxydammara-24-en-3-one.

A total of 17 compounds were identified in CR. Among them, saccharides (**P4** fructose, **P6** sucrose, **P7** raffinose, **P8** stachyose, and **P14** verbascose) and alkaloids (**P5** trigonelline and **P25** codonopsine) were characteristic components [11, 12]. Saccharides showed [M-H]^−^ in the negative ion mode and [M + NH_4_]^+^/[M + Na]^+^in the positive ion mode. The successive neutral loss of hexose (−162 Da) and H_2_O (−18 Da) was used for identification. The typical fragmentation pattern of **P14** verbascose is drawn in Figure 4(a). Alkaloid **P5** trigonelline produced a [M + H]^+^ ion at *m/z* of 138.0546 and had fragment ions at *m/z* of 94, 92, and 78, which correspond to [M - CO_2_ + H]^+^, C_6_H_6_N^+^, and C_5_H_4_N^+^, respectively. P25 codonopsine showed [M + H]^+^ ion at *m/z* of 268.1546, and the fragment ions at *m/z* of 161, 88, and 58 were produced by penta-heterocycle cracking. The typical fragmentation pathways of **P5** trigonelline are drawn in Figure 4(b).

#### 3.2.2. Notoginseng Radix et Rhizoma-Derived Compounds

About 9 compounds were identified in Notoginseng Radix et Rhizoma, and all of the compounds were triterpenoid saponins (**P55** notoginsenoside E, **P58** ginsenoside Re, **P59** ginsenoside Rg1, **P72** ginsenoside Rb1, **P74** notoginsenoside R2, **P76** 20s-ginsenoside Rh1, **P77** ginsenoside Rh4/Rk3, **P82** ginsenoside Rd, and **P91** ginsenoside F2) [13–15].

The neutral loss of Glc (162 Da) and Rha (146 Da) was characteristically appeared in saponin compounds. **P59** ginsenoside Rg1 is taken as example, and it had the [M + HCOO]^−^ ion at *m/z* of 845.4899 and [M + H]^+^ ion at *m/z* of 801.4983. The characteristic product ions at *m/z* of 621 [M-Glc-H_2_O]^+^, 603 [M-Glc-2H_2_O]^+^, 441 [M-2Glc-2H_2_O]^+^, 423 [M-2Glc-3H_2_O]^+^, and 405 [M-2Glc-4H_2_O]^+^ were observed. The typical fragmentation pathways of **P59** ginsenoside Rg1 are drawn in Figure 4(c).

#### 3.2.3. Bletillae Rhizoma-Derived Compounds

A total of 5 characteristic compounds were detected in Bletillae Rhizoma. **P47** dactylorhin A [16], **P56** gymnoside III, and **P61** militarine [17] were structurally similar to that contained two molecules of gastrodin (P22). Neutral loss of Glc (162 Da), H_2_O (18 Da), and gastrodin (268 Da) was used for identification. **P47** dactylorhin A showed the [M - H]^−^ ion at *m/z* of 887.3181 and [M + NH_4_]^+^ ion at *m/z* of 906.3601, while it had characteristic fragment ion at [M-Glc-H_2_O–H]^−^ at *m/z* of 707, [M-gastrodin-H]^−^ at *m/z* of 619, [M-gastrodin-Glc- H_2_O–H]^−^ at *m/z* of 439, [M-gastrodin-Glc + H]^+^ at *m/z* of 459, [gastrodin]^+^ at *m/z* of 269, and [gastrodin-Glc]^+^ at *m/z* of 107. The typical fragmentation pathways of **P47** dactylorhin A are drawn in Figure 4(d).

#### 3.2.4. Sophorae Flos-Derived Compounds

Thirteen compounds were isolated from Sophorae Flos [18–20], and more than half of them were flavonoids, or specifically flavonols (**P37** quercetin 3-O-glucosyl-rutinoside [21], **P39** manghaslin [22], **P43** rutin [23–25], **P45** isoquercitrin [26], **P48** nicotiflorin [27], **P50** narcissin [24, 28], **P70** quercetin [23, 29, 30], and **P79** isorhamnetin [22]). In negative mode, flavonoid glycosides were trend to neutral loss of glycosides. In addition, neutral losses of CH3 (15 Da), CO (28 Da), and RDA cracking could also be observed. **P43** rutin was a vital constituent of Sophorae Flos. It had the [M + H]^+^ ion at *m/z* of 611.1607 and gave characteristic fragment ions at *m/z* of 465 and 303 by successive loss of Glc (162 Da) and Rha (146 Da). The typical fragmentation pathways of **P43** rutin are drawn in Figure 4(e).

#### 3.2.5. Glycyrrhizae Radix et Rhizoma-Derived Compounds

A total of 23 compounds were discriminated in Glycyrrhizae Radix et Rhizoma, and 14 of them were flavonoids (**P44** licuraside/liquiritin apioside, **P46** liquiritin, **P54** naringenin-7-O-glucoside, **P60** violanthin, **P67** pallidiflorin, **P69** isoliquiritigenin [31–33], **P63** licorice glycoside B/D1, **P64** licorice glycoside C2, **P66** licorice glycoside E, **P75** naringenin [34], **P53** choerospodin [35], **P62** ononin/ononin isomer [36], **P90** glyasperin C [37],and **P93** sophoraisoflavone A/semilicoisoflavone B [38]). Different from sophorae, the flavonoids in glycyrrhiza were more abundant, including chalcone, flavones, and flavanones. However, the primary cracking patterns such as neutral loss of glycosides were similar. In addition to flavonoids, triterpenoid saponins were characteristic components as well. Representative compound licorice saponin A3 (P73, [M - H]^−^ at *m/z* of 983.4455, [M + H]^+^ at *m/z* of 985.4644) observed fragments ions at [M-GlcA + H]^+^ at *m/z* of 809, [M-Glc-GlcA + H]^+^ at *m/z* of 647, [M-2GlcA-H_2_O + H]^+^ at *m/z* of 615, [M-Glc-2GlcA + H]^+^ at *m/z* of 471, and [M-Glc-2GlcA-H_2_O + H]^+^ at *m/z* of 453. The fragmentation pathways were similar to P59 drawn in Figure 4(c).

#### 3.2.6. Cynanchi Paniculati Radix et Rhizoma-Derived Compounds

Only one special saponin (steroidal glycoside), namely paniculatumoside A or B (**P89**) [39], was identified in Cynanchi Paniculati Radix et Rhizoma. The cracking mainly occurred at A (*m/z* of 331) and A' rings (*m/z* of 145, 113). The typical fragmentation pathways of **P89** are drawn in Figure 4(f).

#### 3.2.7. Typhae Pollen-Derived Compounds

In this experiment, the characteristic components detected in Typhae Pollen were flavonoids (**P42** typhaneoside [40], **P43** rutin [23–25], **P49** isorhamnetin-3-O-rutinoside-7-O-rhamnoside [24], **P50** narcissin [24, 28], and **P52** isorhamnetin-3-O-beta-galactoside [40]) and carboxylic acids (**P9** L-malic acid [23, 40], **P10** citric acid [40], **P18** succinic acid [40], **P27** 3,4-dihydroxybenzoic acid, **P51** vanillic acid [23], and **P68** decanedioic acid [41]).

Typhaneoside (P42), [M-H]^−^ at *m/z* of 769.2194, [M +H ]^+^ at *m/z* of 771.2327) was a flavonol, and fragment ions were observed after successive loss of Rha (146 Da) and Glc (162 Da). The fragmentation pathways were similar to P43 drawn in Figure 4(e). Simple carboxylic acids were generally responded in the negative mode, and neutral loss of·CH3 (15 Da), H2O (-18 Da), and CO_2_ (-44 Da) was the most usual fragments.

#### 3.2.8. Chebulae Fructus-Derived Compounds

In Chebulae Fructus, gallic acid structure was found in carboxylic acids (**P13** chebulic acid [42], **P23** gallic acid [23, 43], **P26** 5-galloylshikimic acid [44], **P33** brevifolincarboxylic acid [45], and **P40** 3,4,8,9,10-pentahydroxydibenzo[b,d]pyran-6-one [44]), while ellagic acid (gallic acid dimer) structure was tannins (**P28** hamamelitannin [46], **P29** 1,6-di-O-galloyl-*β*-D-glucose [47], **P34** chebulanin(1-O-galloyl-2,4-O-chebuloyl-b-D-Glc [44]), **P36** corilagin [48], and **P41** chebulagic acid [46]). Thus, ellagic acid fragment (*m/z* of 300) and neutral loss of gallic acid (170 Da) could be generally observed. The typical fragmentation pathways of **P36** corilagin are drawn in Figure 4(g).

#### 3.2.9. Atractylodis Macrocephalae Rhizoma-Derived Compounds

The characteristic compound in Atractylodis Macrocephalae Rhizoma was lactone (**P92** atractylenolide III [11, 49]). Lactone was generally responded in the positive mode. Atractylenolide III (P92, [M + H]^+^ at *m/z* of 249.1487) showed fragment ions at [M-H_2_O + H]^+^ at *m/z* of 231, [M-H_2_CO_2_+H]^+^ at *m/z* of 185, [M-C_3_H_4_O + H]^+^ at *m/z* of 175, C_10_H_10_O_2_^+^ at *m/z* of 163, and C_6_H_7_^+^ at *m/z* of 79. The typical fragmentation pathways of **P92** atractylenolide III are drawn in Figure 4(h).

#### 3.2.10. Coicis Semen-Derived Compounds

A total of 15 compounds could be attributed to coicis semen, including 10 carboxylic acid (**P9** L-malic acid [23, 40], **P23** gallic acid [23, 43], **P51** vanillic acid [23], **P95** pseudolaroside B, **P96** quinic acid [23], **P97** protocatechuic acid [50], **P98** caffeic acid [50], **P99** nonanedioic acid [51], **P100** 1-caffeoylquinic acid [52], and **P101** 3-O-feruloylquinic acid [52]), 3 flavonoids (**P43** rutin [23–25], **P70** quercetin [23, 29, 30],and **P103** kaempferol [29, 50]), 1 phenylpropanoid (**P19** p-coumaric acid [11, 53]), and 1 nucleoside (**P102** adenosine [53]).

#### 3.2.11. Ailanthi Cortex-Derived Compounds

In Ailanthi Cortex, 4 compounds were attributed: briefly, 2 flavonoids (**P70** quercetin [23, 29, 30] and **P103** kaempferol [29, 50]), 1 carboxylic acid (**P99** nonanedioic acid [51]), and 1 terpene (**P104** 20-R-hydroxydammara-24-en-3-one). However, only P104 was characteristic, and it had the [M + H]^+^ ion at *m/z* of 443.3881 and gave fragment ions at *m/z* of 425 by neutral loss of H_2_O (18 Da). The crack of C ring formed ions at *m/z* of 221 and 207. The typical fragmentation pathways of **P104** are drawn in Figure 4(i).

### 3.3. Characterization of LCD-Related Xenobiotics in Rat Biological Samples

**Figure 5:**
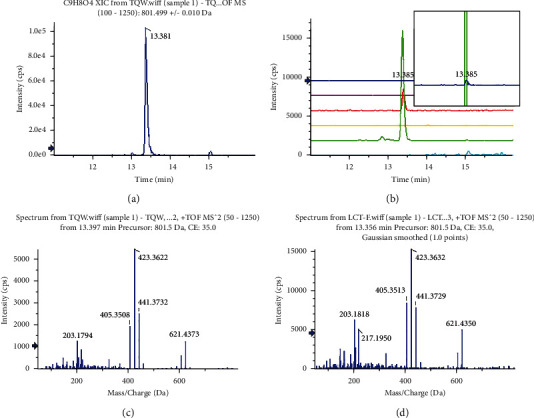
Identification of prototypes in bio-samples, and P59 ginsenoside Rg1 is taken as an example. (a) XIC of ginsenoside Rg1 in LCD; (b) multiple XICs of ginsenoside Rg1 in bio-samples. From top to bottom: administration plasma, blank plasma, administration urine, blank urine, administration feces, and blank feces. Ginsenoside Rg1 showed the highest intensity in feces, lowest in plasma, and no response in blank samples; (c) MS/MS spectrum of ginsenoside Rg1 in LCD; (d) MS/MS spectrum of ginsenoside Rg1 in feces.

According to the compound characterization of LCD, the fragmentation patterns of mass spectrometry (accurate molecular weight and secondary debris) and retention time of chromatography were adopted to analyze the components in plasma, urine, and feces. **P59** ginsenoside Rg1 is taken as example, as shown in the XIC of LCD (Figure 5(a)) and multiple XICs of 6 bio-samples (Figure 5(b)), and a peak at 13.4 min was clearly observed in administration of bio-samples but not in the blanks. Importantly, the MS/MS spectra (*m/z* of 621, 441, 423, 405, and 203) of ginsenoside Rg1 in LCD (Figure 5(c)) and bio-samples (Figure 5(d)) were similar.

Based on the above principles, a total of 50 components were matched in biological samples, and these components would play a key role in explaining the mechanism of LCD in the future. In particular, flavonoids (**P43**, **P46**, and **P50**) and saponins (**P55** and **P72**) deserved higher attention as the five components were observed in all three bio-samples besides that were common to organisms (**P1**, **P11**, **P15**, **P24**, **P31**, and **P68**). In addition, 12 compounds were just observed in the fecal sample, mainly including some alkaloids (**P25** and **P65**), flavonoids (**P37**, **P42**, **P45**, **P52**, and **P70**), saponin (**P74**), and other small molecules (**P6**, **P9**, **P35**, and **P40**). These compounds may not be absorbed into the blood, but are still effective in regulating gut microbiota. The detailed information about the distribution of components in plasma, urine, and feces is summarized in Table 2.

**Figure 6:**
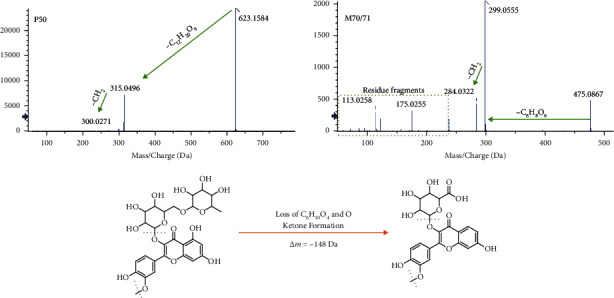
Identification of metabolites in bio-samples.

**Figure 7:**
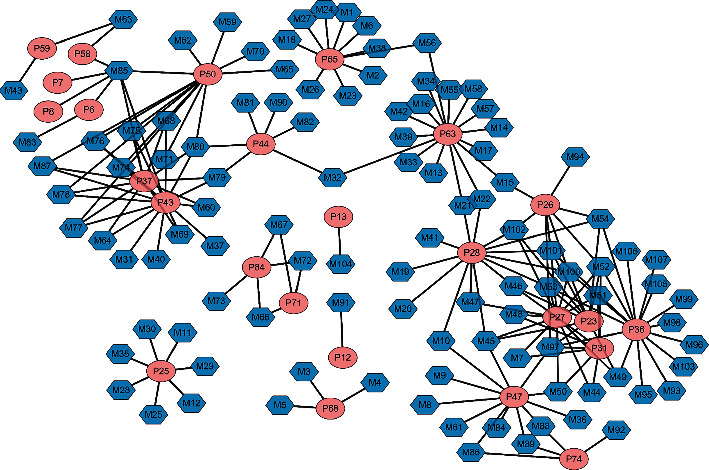
Correlation between prototype and metabolites.

Furtherly, the phase I and phase II metabolic regularity, as well as the similarity of secondary mass spectrum profile, was used to identify the metabolite. Those metabolites were annotated through automatic matching with prototype components by MetabolitePilot Software. Briefly, MetabolitePilot operated prototype-metabolite matching through mass defect filter (MDF), characteristic product ion filter (PIF), and neutral loss filter (NLF). As shown in Figure 6, the mass defect from P50 to M70/71 was -148 Da with the biotransformation named “loss of C_6_H_10_O_4_ and O (hydrolysis, phase I) + ketone formation (phase I).” Furthermore, neutral loss of glycosides and methylene was both observed in the MS/MS spectra of P50 and M70/71, which implied the similar skeleton. That was to say, these compounds were structurally related, and M70/71 could be the metabolites of P50. As a result, a total of 107 metabolites were matched with 25 prototypes in plasma, urine, or feces. The network of prototype-metabolite matching is drawn as in Figure 7. The details involving the distribution and biotransformations of metabolites are listed in Table 3. It was worth noting that although some prototypes have not been observed in bio-samples, they still are effective through metabolites. For example, P28 hamamelitannin produced 14 metabolites that were all detected in urine, and 5 were found in plasma and 2 in feces. It could be metabolized in the gut, and metabolites were furtherly absorbed into the bloodstream. In total, 29 prototype components and 22 metabolites were detected in plasma. About 27 prototypes and 96 metabolites were detected in urine, and 34 prototypes and 18 metabolites were detected in feces. These substances were considered to constitute the pharmacodynamic substance basis of LCD.

**P2** arginine [54–56], **P5** trigonelline [57], **P59** ginsenoside Rg1 [58], **P69** isoliquiritigenin [59], **P82** ginsenoside Rd [60, 61], and **P84** glycyrrhizic acid [62–64] would alleviate the symptom of UC based on anti-inflammation or antioxidant activities. Besides, **P15** isoleucine [65], **P17** uridine [12, 66], **P21** guanosine [67], **P23** gallic acid [68, 69], **P43** rutin [70, 71], **P51** vanillic acid [72], **P70** quercetin [73, 74], **P72** ginsenoside Rb1 [75], and **P81** betulin [76]were confirmed to treat UC through NF-*κ*B pathway. **P8** stachyose increased beneficial microbiota and bacterial diversity to alleviate colitis mice [77]. **P45** hyperoside ameliorates ulcer colitis mice through MKRN1-mediated regulation of PPAR*γ* signaling and Th17/Treg balance [78]. The effect of those metabolisms on UC was worth to study for new drug development.

## Figures and Tables

**Table 1:** Chemical component of LCD.

	Alkaloid	Amino acid	Oligosaccharides	Saponins	Lignans	Flavonoids	Organic acids	Tannins	Others (Nucleosides, glycosides, esters, etc.)	Total
CR	2	4	5	—	—	—	—	—	6 (3)	17
NRR	—	—	—	9	—	—	—	—	—	9
BR	—	—	—	—	3	—	1	—	1	5
SF	—	1	—	2	—	8 (3)	—	—	2	13
GRR	—	—	—	8	—	14	—	—	1	23
CPRR	—	—	—	1	—	—	—	—	—	1
TP	—	3	—	—	—	5 (2)	6 (2)	—	2 (1)	16
CF	—	—	—	—	—	—	5 (1)	5	1	11
AMR	—	—	—	—	—	—	1	—	2 (1)	3
CS	—	—	—	—	—	3 (3)	10 (4)	—	2 (1)	15
AC	—	—	—	—	—	2 (2)	1 (1)	—	1	4
Total	2	8	5	20	3	26	20	5	15	104

The number in the brackets was the repeat compounds.

**Table 2:** Identification of the major components present in LCD by UPLC-QTOF-MS.

No.	Compound	Formula	Rt (min)	Ion mode	Cal *m/z*	ESI-*m/z*	ppm	Fragment ions (*m/z*)	Ion mode	Cal *m/z*	ESI+*m/z*	ppm	Fragment ions (*m/z*)	Compound class	Source	Reference
P1	Choline	C_5_H_13_NO	1.25	—	—	—	—	—	[M + H]^+^	104.1070	104.1065	−4.8	60, 59	Choline	CR	[11]
P2	Arginine	C_6_H_14_N_4_O_2_	1.21	[M - H]^−^	173.1039	173.1040	0.6	173.131	[M + H]^+^	175.1190	175.1189	−0.6	130, 116, 70, 60	Amino acid	CR	[11]
P3	Asparagine	C_4_H_8_N_2_O_3_	1.24	[M - H]^−^	131.0457	131.0462	3.8	114, 113, 95, 72.58	[M + H]^+^	133.0608	133.0606	−1.5	74	Amino acid	CR	[11]
P4	Fructose	C_6_H_12_O_6_	1.33	[M - H]^−^	179.0556	179.0555	−0.6	161, 131, 101, 85, 59	[M + Na]^+^	203.0526	203.0524	−1.0	158.88.70	Saccharides	CR	[11]
P5	Trigonelline^*∗*^	C_7_H_7_NO_2_	1.36	—	—	—	—	—	[M + H]^+^	138.0550	138.0546	−2.9	94, 92, 78,	Alkaloids	CR	[11]
P6	Sucrose	C_12_H_22_O_11_	1.43	[M - H]^−^	341.1089	341.1089	0.0	179, 89	—	—	—	—	—	Saccharides	CR	[11]
P7	Raffinose	C_18_H_32_O_16_	1.51	[M - H]^−^	503.1618	503.1606	−2.4	323, 191, 179	[M + NH4]+	522.2029	522.2019	−1.9	325, 289, 163, 145, 127	Saccharides	CR	[11]
P8	Stachyose	C_24_H_42_O_21_	1.65	[M - H]^−^	665.2146	665.2133	−2.0	341, 323, 179, 161	[M + NH4]+	684.2557	684.2549	−1.2	487, 325, 289, 163, 145, 127	Saccharides	CR	[11]
P9	L-Malic acid	C_4_H_6_O_5_	1.66	[M - H]^−^	133.0142	133.0142	0.0	115, 89, 71	—	—	—	—	—	Carboxylic acids	TP/CS	[23, 40]
P10	Citric acid	C_6_H_8_O_7_	1.69	[M - H]^−^	191.0197	191.0200	1.6	111, 85, 73	—	—	—	—	—	Carboxylic acids	TP	[40]
P11	Valine	C_5_H_11_NO_2_	1.69	—	—	—	—	—	[M + H]^+^	118.0863	118.0854	−7.6	72, 55	Amino acid	TP	[41]
P12	Adenine nucleoside	C_10_H_13_N_5_O_4_	1.76/3.20	—	—	—	—	—	[M + H]^+^	268.1040	268.1038	−0.7	136, 119	Nucleoside	CR	[11]
P13	Chebulic acid^*∗*^	C_14_H_12_O_11_	1.80/2.27	[M - H]^−^	355.0307	355.0296	−3.1	337, 293, 249, 205	—	—	—	—	—	Carboxylic acids	CF	[42]
P14	Verbascose	C_30_H_52_O_26_	2.00	[M - H]^−^	827.2669	827.2674	0.6	665, 503, 341, 179, 161	[M + Na]^+^	851.2639	851.2618	−2.5	689	Saccharides	CR	[11]
P15	Isoleucine	C_6_H_13_NO_2_	2.07	—	—	—	—	—	[M + H]+	132.1019	132.1013	−4.5	86, 85	Amino acid	CR	[11]
P16	L-Pyroglutamic acid	C_5_H_7_NO_3_	2.41	[M - H]^−^	128.0348	128.0353	3.9	82	[M + H]+	130.0499	130.0493	−4.6	84.56	Amino acid	CR	[11]
P17	Uridine	C_9_H_12_N_2_O_6_	2.66	[M - H]^−^	243.0623	243.0623	0.0	200, 152, 110	[M + H]+	245.0768	245.0770	0.8	113, 70	Nucleoside	TP	[12]
P18	Succinic acid	C_4_H_6_O_4_	2.70	[M - H]^−^	117.0193	117.0192	−0.9	73	—	—	—	—	—	Carboxylic acids	TP	[40]
P19	p-Coumaric acid	C_9_H_8_O_3_	2.86	—	—	—	—	—	[M + H]+	165.0546	165.0541	−3.0	162.123.77	Phenylpropanoids	CR/CS	[11, 53]
P20	Leucine	C_6_H_13_NO_2_	3.10	—	—	—	—	—	[M + H]+	132.1019	132.1014	−3.8	86	Amino acid	TP	[41]
P21	Guanosine	C_10_H_13_N_5_O_5_	3.74	[M - H]^−^	282.0838	282.0841	1.1	150, 133, 107	—	—	—	—	—	Nucleoside	CR/TP	[11, 12]
P22	Gastrodin	C_13_H_18_O_7_	3.85	—	—	—	—	—	[M + NH4]+	304.1391	304.1396	1.6	108, 107, 105	Glycoside	BR	[17]
P23	Gallic acid^*∗*^	C_7_H_6_O_5_	4.17	[M - H]^−^	169.0142	169.0146	2.4	125	[M + H]^+^	171.0288	171.0281	−4.1	153, 107	Carboxylic acids	CF/CS	[23, 43]
P24	Phenylalanine	C_9_H_11_NO_2_	5.12	[M - H]^−^	164.0717	164.0718	0.6	147, 103, 72	[M + H]^+^	166.0863	166.0859	−2.4	120, 103, 77	Amino acid	TP	[41]
P25	Codonopsine	C_14_H_21_NO_4_	6.27	—	—	—	—	—	[M + H]^+^	268.1543	268.1546	1.1	161, 121, 88, 58	Alkaloids	CR	[11]
P26	5-Galloylshikimic acid	C_14_H_14_O_9_	6.74	[M - H]^−^	325.0565	325.0570	1.5	169, 125	—	—	—	—	—	Carboxylic acids	CF	[44]
P27	3, 4-Dihydroxybenzoic acid	C_7_H_6_O_4_	6.93	[M - H]^−^	153.0193	153.0197	2.6	109, 108	—	—	—	—	—	Carboxylic acids	TP	—
P28	Hamamelitannin	C_20_H_20_O_14_	7.95	[M - H]^−^	483.0780	483.0773	−1.4	271, 211, 169, 125	—	—	—	—	—	Tannins	CF	[46]
P29	1, 6-Di-O-galloyl-*β*-D-glucose	C_20_H_20_O_14_	8.52/8.91/9.09/9.25	[M - H]^−^	483.0780	483.0779	−0.2	423, 271, 211, 169	—	—	—	—	—	Tannins	CF	[47]
P30	5-Hydroxyferulic acid	C_10_H_10_O_5_	8.99	[M - H]^−^	209.0456	209.0461	2.4	165, 121, 59	—	—	—	—	—	Carboxylic acids	AMR	-
P31	4-Hydroxybenzoic acid	C_7_H_6_O_3_	9.16	[M - H]^−^	137.0244	137.0241	−2.2	93	—	—	—	—	—	Carboxylic acids	BR	[16]
P32	Soyamaloside C	C_23_H_32_O_16_	9.61	[M - H]^−^	563.1618	563.1614	−0.7	461, 419	—	—	—	—	—	Glycoside	SF	[19]
P33	Brevifolincarboxylic acid	C_13_H_8_O_8_	9.73	[M - H]^−^	291.0141	291.0147	2.1	247, 219, 191	—	—	—	—	—	Carboxylic acids	CF	[45]
P34	Chebulanin(1-O-galloyl-2, 4-O-chebuloyl-b-D-Glc)	C_27_H_24_O_19_	9.98	[M - H]^−^	651.0834	651.0839	0.8	633, 481, 275, 169	—	—	—	—	—	Tannins	CF	[44]
P35	6, 7-Dihydroxycoumarin^*∗*^	C_9_H_6_O_4_	10.04	[M - H]^−^	177.0188	177.0192	2.3	177, 133, 105, 89	—	—	—	—	—	Coumarins	GRR	[34]
P36	Corilagin^*∗*^	C_27_H_22_O_18_	10.18	[M - H]^−^	633.0704	633.0732	4.4	463, 300, 169	[M + NH4]+	652.1145	652.1140	−0.7	465, 363, 303, 277	Tannins	CF	[48]
P37	Quercetin 3-O-glucosyl-rutinoside	C_33_H_40_O_21_	10.37	[M - H]^−^	771.1993	771.1990	−0.4	300	[M + H]^+^	773.2136	773.2134	−0.3	465, 303	Flavonoids	SF	[21]
P38	Euphormisin M3	C_27_H_24_O_18_	10.55	[M - H]^−^	635.0890	635.0891	0.2	483, 465, 169, 125	[M + NH4]+	654.1301	654.1280	−3.3	467, 297, 171, 153	Glycoside	CF	[46]
P39	Manghaslin	C_33_H_40_O_20_	10.58	[M - H]^−^	755.2040	755.2041	0.1	609, 447, 299	[M + H]^+^	757.2187	757.2179	−1.0	661, 449, 303	Flavonoids	SF	[22]
P40	3,4,8,9,10-Pentahydroxydibenzo[b,d]pyran-6-one	C_13_H_8_O_7_	10.96	[M - H]^−^	275.0192	275.0201	3.3	258, 257, 229, 201, 173, 145,	—	—	—	—	—	Carboxylic acids	CF	[44]
P41	Chebulagic acid	C_41_H_30_O_27_	11.05	[M - H]^−^	953.0896	953.0903	0.7	301, 275	—	—	—	—	—	Tannins	CF	[46]
P42	Typhaneoside^*∗*^	C_34_H_42_O_20_	11.25	[M - H]^−^	769.2197	769.2194	−0.4	623, 314, 189	[M + H]^+^	771.2343	771.2327	−2.1	625, 479, 317	Flavonoids	TP	[40]
P43	Rutin^*∗*^	C_27_H_30_O_16_	11.56	[M - H]^−^	609.1461	609.1459	−0.3	301	[M + H]^+^	611.1607	611.1607	−0.1	465, 303, 85, 71	Flavonoids	SF/TP/CS	[23–25]
P44	Licuraside/liquiritin apioside	C_26_H_30_O_13_	11.65	[M - H]^−^	549.1608	549.1611	0.5	255,135	[M + H]^+^	551.1765	551.1741	−4.4	257,137	Flavonoids	GRR	[31]
P45	Hyperoside^*∗*^	C_21_H_20_O_12_	11.87	[M - H]^−^	463.0882	463.0869	−2.8	300, 301	[M+H]^+^	465.1028	465.1021	−1.5	303	Flavonoids	SF	[26]
P46	Liquiritin^*∗*^	C_21_H_22_O_9_	11.85	[M - H]^−^	417.1191	417.1184	−1.7	255, 135	[M + NH4]^+^	436.1603	436.1592	−2.5	257, 137	Flavonoids	GRR	[31]
P47	Dactylorhin A	C_40_H_56_O_22_	12.06	[M - H]^−^	887.3190	887.3181	−1.0	707, 619, 439	[M + NH4]^+^	906.3603	906.3601	−0.3	621, 537, 459, 431, 403, 375, 325, 297, 269, 213, 191, 107	Lignans	BR	[16]
P48	Nicotiflorin^*∗*^	C_27_H_30_O_15_	12.18	[M - H]^−^	593.1512	593.1502	−1.7	285	[M + H]^+^	595.1658	595.1657	−0.2	449, 431, 287	Flavonoids	SF	[27]
P49	Isorhamnetin-3-O-rutinoside-7-O-rhamnoside	C_34_H_40_O_20_	12.23	[M - H]^−^	767.2040	767.2046	0.8	705, 665, 623, 314, 299, 271, 179	—	—	—	—	—	Flavonoids	TP	[24]
P50	Narcissin	C_28_H_32_O_16_	12.27	[M - H]^−^	623.1618	623.1600	−2.9	315, 314, 300, 285, 271, 255, 151	[M + H]^+^	625.1763	625.1754	−1.4	317	Flavonoids	TP/SF	[24, 28]
P51	Vanillic acid	C_8_H_8_O_4_	12.60	[M - H]^−^	167.0350	167.0347	−1.8	152,108	—	—	—	—	—	Carboxylic acids	TP/CS	[23]
P52	Isorhamnetin-3-O-beta-galactoside	C_22_H_22_O_12_	12.70	[M - H]^−^	477.1038	477.1027	−2.3	314, 285	[M + H]^+^	479.1184	479.1168	−3.3	317	Flavonoids	TP	[40]
P53	Choerospodin	C_21_H_22_O_10_	12.83	[M - H]^−^	433.1140	433.1151	2.5	271, 151	[M + H]^+^	435.1286	435.1268	−4.1	273, 153	Flavonoids	GRR	[35]
P54	Naringenin-7-O-glucoside	C_21_H_22_O_10_	12.95	[M - H]^−^	433.1140	433.1151	2.5	433, 271, 151	[M + H]^+^	435.1286	435.1274	−2.8	153,147	Flavonoids	GRR	[31]
P55	Notoginsenoside E	C_48_H_82_O_20_	12.96	[M - H]^−^	977.5321	977.5308	−1.3	931	—	—	—	—	—	Saponins	NRR	[13]
P56	Gymnoside III	C_42_H_58_O_23_	13.22	[M - H]^−^	975.3351	975.3336	−1.5	707, 661, 439	[M + NH4]^+^	948.3709	948.3692	−1.8	825, 663, 635, 501, 473, 395, 367, 297, 205, 107	Lignans	BR	—
P57	Lobetyolin^*∗*^	C_20_H_28_O_8_	13.24	—	—	—	—	—	[M + NH4]^+^	414.2124	414.2121	−0.6	199, 155	Glycoside	CR	[11]
P58	Ginsenoside Re^*∗*^	C_48_H_82_O_18_	13.34/15.12/16.09/16.51	[M + COOH]^−^	991.5483	991.5459	−2.4	783, 621	[M + H]^+^	947.5577	947.5544	−3.5	767, 749, 605, 587, 443, 407, 325	Saponins	NRR	[14]
P59	Ginsenoside Rg1^*∗*^	C_42_H_72_O_14_	13.40	[M + COOH]^−^	845.4904	845.4899	−0.6	799, 637	[M + H]^+^	801.4998	801.4983	−1.8	621, 603, 441, 423, 405, 325	Saponins	NRR	[14]
P60	Violanthin	C_27_H_30_O_14_	13.65	[M - H]^−^	577.1563	577.1553	−1.7	515, 475, 433, 145	[M+H]^+^	579.1708	579.1701	−1.2	453, 291, 147	Flavonoids	GRR	[31]
P61	Militarine	C_34_H_46_O_17_	13.65	[M + COOH]^−^	771.2717	771.2702	−1.9	725, 457, 285, 153	[M + NH4]^+^	744.3075	744.3069	−0.8	107	Lignans	BR	[17]
P62	Ononin/ononin isomer	C_22_H_22_O_9_	13.73	—	—	—	—	—	[M + H]^+^	431.1342	431.1337	−1.2	269	Flavonoids	GRR	[36]
P63	Licorice glycoside B/D1	C_35_H_36_O_15_	13.73	[M - H]^−^	695.1981	695.1961	−2.9	255, 399, 531, 549	—	—	—	—	—	Flavonoids	GRR	[34]
P64	Licorice glycoside C2	C_36_H_38_O_16_	13.81	[M - H]^−^	725.2087	725.2076	−1.5	549, 531, 255, 193	[M + H]^+^	727.2233	727.2233	0.0	309,297,245	Flavonoids	GRR	[34]
P65	N, N′-diferuloylputrescine	C_24_H_28_N_2_O_6_	14.17	[M - H]^−^	439.1875	439.1885	2.3	289, 149	[M + H]^+^	441.2020	441.2009	−2.5	265, 177	Amino acid	SF	[18]
P66	Licorice glycoside E	C_35_H_35_NO_14_	14.34	[M - H]^−^	692.1985	692.1983	−0.3	549, 531	[M + H]^+^	694.2130	694.2114	−2.3	240, 144	Flavonoids	GRR	[34]
P67	Pallidiflorin	C_16_H_12_O_4_	14.42	[M - H]^−^	267.0663	267.0661	−0.7	267, 252, 195, 132	—	—	—	—	—	Flavonoids	GRR	[31]
P68	Decanedioic acid	C_10_H_18_O_4_	14.45	[M - H]^−^	201.1132	201.1125	−3.5	183, 139,	—	—	—	—	—	Carboxylic acids	TP	[41]
P69	Isoliquiritigenin	C_15_H_12_O_4_	14.46	[M - H]^−^	255.0663	255.0655	−3.1	255, 135, 119, 91	[M + H]^+^	257.0808	257.0816	3.1	257, 147, 137, 119, 81	Flavonoids	GRR	[31]
P70	Quercetin^*∗*^	C_15_H_10_O_7_	14.67	[M - H]^−^	301.0354	301.0346	−2.7	179, 151	[M + H]^+^	303.0499	303.0503	1.3	245, 301, 106, 151	Flavonoids	SF/CS/AC	[23, 29, 30]
P71	Licorice saponin A3	C_48_H_72_O_21_	14.69	[M - H]^−^	983.4493	983.4455	−3.9	821, 645, 351	[M + H]^+^	985.4642	985.4644	0.3	809, 647, 615, 471, 453	Saponins	GRR	[31]
P72	Ginsenoside Rb1^*∗*^	C_54_H_92_O_23_	15.13	[M + HCOOH–2H]2-	599.2997	599.2987	−1.7	1107, 945, 783, 553, 161	[M + H]^+^	1109.6106	1109.6078	−2.5	767, 649, 605, 487, 425, 407, 325, 289	Saponins	NRR	[14]
P73	Licorice saponin G2	C_42_H_62_O_17_	15.24	[M - H]^−^	837.3914	837.3898	−1.9	351	[M + H]^+^	839.4062	839.4046	−1.9	839, 663, 487, 469	Saponins	GRR	[31]
P74	Notoginsenoside R2	C_41_H_70_O_13_	15.31	[M + COOH]^−^	815.4799	815.4787	−1.5	769, 637	—	—	—	—	—	Saponins	NRR	[13]
P75	Naringenin^*∗*^	C_15_H_12_O_5_	15.57	[M - H]^−^	271.0604	271.0612	3.0	151, 119	[M + H]^+^	273.0757	273.0760	1.1	153, 147	Flavonoids	GRR	[34]
P76	20S-Ginsenoside Rh1^*∗*^	C_36_H_62_O_9_	15.71	[M + COOH]^−^	683.4376	683.4359	−2.5	673, 475	—	—	—	—	—	Saponins	NRR	[15]
P77	Ginsenoside Rh4/Rk3	C_36_H_60_O_8_	15.76	—	—	—	—	—	[M + H]^+^	621.4364	621.4361	−0.4	441, 423, 405, 221, 203, 187	Saponins	NRR	[15]
P78	Licorice saponin G2 isomer	C_42_H_62_O_17_	15.83	[M - H]^−^	837.3914	837.3901	−1.6	351	[M + H]^+^	839.4062	839.4065	0.3	839, 663, 645, 487, 469	Saponins	GRR	[31]
P79	Isorhamnetin^*∗*^	C_16_H_12_O_7_	15.95	—	—	—	—	—	[M + H]^+^	317.0656	317.0659	0.9	302, 153	Flavonoids	SF	[22]
P80	Raho glycyrrhizin	C_48_H_72_O_20_	15.96	[M - H]^−^	967.4544	967.4517	−2.8	329	[M + H]^+^	969.4692	969.4650	−4.4	621, 453, 435, 405, 217	Saponins	GRR	[32]
P81	Betulin	C_30_H_50_O_2_	16.10	[M - H]^−^	—	—	—	—	[M + H]^+^	443.3884	443.3886	0.5	443, 425, 407, 271, 207, 175, 59	Triterpenoids	SF	[20]
P82	Ginsenoside Rd^*∗*^	C_48_H_82_O_18_	16.11	[M + COOH]^−^	991.5483	991.5459	−2.4	783, 621	—	—	—	—	—	Saponins	NRR	[13]
P83	Yunganoside G1	C_48_H_74_O_21_	16.14	—	—	—	—	—	[M + H]^+^	987.4798	987.4779	−1.9	841, 665, 629, 471, 453, 441, 353	Saponins	GRR	[33]
P84	Glycyrrhizic acid^*∗*^	C_42_H_62_O_16_	16.31	[M - H]^−^	821.3965	821.3942	−2.8	759, 351, 193	[M + H]^+^	823.4113	823.4111	−0.2	823, 647, 471, 453, 194	Saponins	GRR	[31]
P85	Glycyrrhizic isomer /uralsaponin A/licorice saponin K2/licorice saponin H2	C_42_H_62_O_16_	16.82/17.02	[M - H]^−^	821.3965	821.3953	−1.5	351, 193	[M + H]^+^	823.4113	823.4111	−0.2	823, 647, 471, 453, 194	Saponins	GRR	[31]
P86	Kaikasaponin III	C_48_H_78_O_17_	17.15	[M + COOH]^−^	971.5221	971.5194	−2.8	925	[M + NH4]^+^	944.5580	944.5553	−2.9	503, 485, 425, 407, 309, 287, 147	Saponins	SF	[19]
P87	Uralsaponin C/licorice saponin J2	C_42_H_64_O_16_	17.22	—	—	—	—	—	[M + H]^+^	825.4270	825.4248	−2.6	825, 613, 455, 409, 397, 317, 177, 159, 141	Saponins	GRR	[31]
P88	Kaikasaponin I	C_42_H_68_O_13_	17.73	—	—	—	—	—	[M + NH4]^+^	798.5001	798.4988	−1.6	425, 407, 339, 163	Saponins	SF	[19]
P89	Paniculatumoside A/paniculatumoside B	C_28_H_40_O_9_	18.00	—	—	—	—	—	[M + H]+	521.2747	521.2739	−1.5	331, 145, 113	Saponins (steroidal glycoside)	CPRR	[39]
P90	Glyasperin C	C_21_H_24_O_5_	18.09	—	—	—	—	—	[M + H]^+^	357.1697	357.1693	−1.1	283, 165, 137, 123	Flavonoids	GRR	[37]
P91	Ginsenoside F2	C_42_H_72_O_13_	18.57	[M - H]^−^	779.4587	779.4575	−1.5	799	[M + Na]^+^	807.4868	807.4835	−4.1	785, 767, 443, 407, 325	Saponins	NRR	[15]
P92	Atractylenolide III	C_15_H_20_O_3_	18.62	—	—	—	—	—	[M + H]^+^	249.1497	249.1487	−4.0	231, 175, 163, 185, 161, 105, 79	Lactone	CR/AMR	[11, 49]
P93	Sophoraisoflavone A/semilicoisoflavone B	C_20_H_16_O_6_	19.91	—	—	—	—	—	[M + H]^+^	353.1020	353.1018	−0.6	335, 311, 299, 215, 199, 153	Flavonoids	GRR	[38]
P94	7-[4-(11-hydroxy-undecyloxy)-phenyl]-7-pyridin-3-yl-hept-6-enoic acid ethyl ester	C_31_H_45_NO_4_	20.82	—	—	—	—	—	[M + H]^+^	496.3421	496.3392	−5.8	478, 184, 104	Esters	AMR	[49]
P95	Pseudolaroside B	C_14_H_18_O_9_	6.73	[M - H]^−^	329.08781	329.0883	1.49	163	—	—	—	—	—	Carboxylic acids	CS	—
P96	Quinic acid	C_7_H_12_O_6_	1.42	[M - H]^−^	191.05611	191.0561	−0.05	191	—	—	—	—	—	Carboxylic acids	CS	[23]
P97	Protocatechuic acid	C_7_H_6_O_4_	6.93	[M - H]^−^	153.01933	153.01936	0.20	109, 91	—	—	—	—	—	Carboxylic acids	CS	[50]
P98	Caffeic acid	C_9_H_8_O_4_	10.13	[M - H]^−^	179.03498	179.0349	−0.45	135	—	—	—	—	—	Carboxylic acids	CS	[50]
P99	Nonanedioic acid	C_9_H_16_O_4_	13.2	[M - H]^−^	187.09758	187.0978	1.18	187, 169, 125, 97, 57	[M + H]^+^	189.1121	189.1122	0.53	171, 125, 97, 55	Carboxylic acids	CS/AC	[51]
P100	1-Caffeoylquinic acid	C_17_H_20_O_9_	9.72	[M - H]^−^	367.10346	367.1027	−2.07	193, 173	[M+H]^+^	369.118	369.1183	0.81	177, 145	Carboxylic acids	CS	[52]
P101	3-O-Feruloylquinic acid	C_17_H_20_O_9_	10.89	[M - H]^−^	367.10346	367.1032	−0.71	193, 191, 173	[M + H]^+^	369.118	369.1183	0.81	177, 145	Carboxylic acids	CS	[52]
P102	Adenosine	C_10_H_13_N_5_O_4_	3.35	—	—	—	—	—	[M + H]^+^	268.104	268.1042	0.75	136	Nucleoside	CS	[53]
P103	Kaempferol	C_15_H_10_O_6_	15.72	—	—	—	—	—	[M + H]^+^	287.055	287.0552	0.70	231, 213, 165, 153, 121	Flavonoids	CS/AC	[29, 50]
P104	20-R-hydroxydammara-24-en-3-one	C_30_H_50_O_2_	16.08	—	—	—	—	—	[M + H]^+^	443.3884	443.3881	−0.68	425, 221, 207, 189, 133	Terpenes	AC	—

^*∗*^: compounds verified by standards

**Table 3:** Prototype and metabolic components of LCD in rat serum, urine, and fecal samples.

Metabolites	Prototype	Component name	Formula	tR (min)	Serum	Urine	Feces
—	P1	Choline	C_5_H_13_NO	1.25	√	√	√
—	P2	Arginine	C_6_H_14_N_4_O_2_	1.21	√	—	√
—	P3	Asparagine	C_4_H_8_N_2_O_3_	1.24	—	—	—
—	P4	Fructose	C_6_H_12_O_6_	1.33	—	—	—
—	P5	Trigonelline	C_7_H_7_NO_2_	1.36	√	√	—
—	P6	Sucrose	C_12_H_22_O_11_	1.43	—	—	√
—	P7	Raffinose	C_18_H_32_O_16_	1.51	—	—	—
—	P8	Stachyose	C_24_H_42_O_21_	1.65	—	—	—
—	P9	L-Malic acid	C_4_H_6_O_5_	1.66	—	—	√
—	P10	Citric acid	C_6_H_8_O_7_	1.69	√	√	—
—	P11	Valine	C_5_H_11_NO_2_	1.69	√	√	√
—	P12	Adenine nucleoside	C_10_H_13_N_5_O_4_	1.76/3.20	√	—	√
—	P13	Chebulic acid	C_14_H_12_O_11_	1.80/2.27	—	—	—
—	P14	Verbascose	C_30_H_52_O_26_	2.00	—	—	—
—	P15	Isoleucine	C_6_H_13_NO_2_	2.07	√	√	√
—	P16	L-Pyroglutamic acid	C_5_H_7_NO_3_	2.41	√	√	—
—	P17	Uridine	C_9_H_12_N_2_O_6_	2.66	—	—	—
—	P18	Succinic acid	C_4_H_6_O_4_	2.70	√	—	√
—	P19	p-Coumaric acid	C_9_H_8_O_3_	2.86	√	—	—
—	P20	Leucine	C_6_H_13_NO_2_	3.10	√	—	—
—	P21	Guanosine	C_10_H_13_N_5_O_5_	3.74	—	—	—
—	P22	Gastrodin	C_13_H_18_O_7_	3.85	—	√	—
—	P23	Gallic acid	C_7_H_6_O_5_	4.17	—	√	√
—	P24	Phenylalanine	C_9_H_11_NO_2_	5.12	√	√	√
—	P25	Codonopsine	C_14_H_21_NO_4_	6.27	—	—	√
—	P26	5-Galloylshikimic acid	C_14_H_14_O_9_	6.74	—	—	—
—	P27	3,4-Dihydroxybenzoic acid	C_7_H_6_O_4_	6.93	—	√	√
—	P28	Hamamelitannin	C_20_H_20_O_14_	7.95	—	—	—
—	P29	1,6-Di-O-galloyl-*β*-D-glucose	C_20_H_20_O_14_	8.52/8.91/9.09/9.25	—	—	—
—	P30	5-Hydroxyferulic acid	C_10_H_10_O_5_	8.99	√	√	—
—	P31	4-Hydroxybenzoic acid	C_7_H_6_O_3_	9.16	√	√	√
—	P32	Soyamaloside C	C_23_H_32_O_16_	9.61	√	—	—
—	P33	Brevifolincarboxylic acid	C_13_H_8_O_8_	9.73	—	—	—
—	P34	Chebulanin(1-O-galloyl-2,4-O-chebuloyl-b-D-Glc)	C_27_H_24_O_19_	9.98	—	—	—
—	P35	6,7-Dihydroxycoumarin	C_9_H_6_O_4_	10.04	—	—	√
—	P36	Corilagin	C_27_H_22_O_18_	10.18	—	—	—
—	P37	Quercetin 3-O-glucosyl-rutinoside	C_33_H_40_O_21_	10.37	—	—	√
—	P38	Euphormisin M3	C_27_H_24_O_18_	10.55	—	—	—
—	P39	Manghaslin	C_33_H_40_O_20_	10.58	—	—	—
—	P40	3,4,8,9,10-Pentahydroxydibenzo[b,d]pyran-6-one	C_13_H_8_O_7_	10.96	—	—	√
—	P41	Chebulagic acid	C_41_H_30_O_27_	11.05	—	—	—
—	P42	Typhaneoside	C_34_H_42_O_20_	11.25	—	—	√
—	P43	Rutin	C_27_H_30_O_16_	11.56	√	√	√
—	P44	Licuraside/liquiritin apioside	C_26_H_30_O_13_	11.65	—	√	√
—	P45	Hyperoside	C_21_H_20_O_12_	11.87	—	—	√
—	P46	Liquiritin	C_21_H_22_O_9_	11.85	√	√	√
—	P47	Dactylorhin A	C_40_H_56_O_22_	12.06	√	√	—
—	P48	Nicotiflorin	C_27_H_30_O_15_	12.18	√	—	√
—	P49	Isorhamnetin-3-O-rutinoside-7-O-rhamnoside	C_34_H_40_O_20_	12.23	—	—	—
—	P50	Narcissin	C_28_H_32_O_16_	12.27	√	√	√
—	P51	Vanillic acid	C_8_H_8_O_4_	12.60	√	√	—
—	P52	Isorhamnetin-3-O-beta-galactoside	C_22_H_22_O_12_	12.70	—	—	√
—	P53	Choerospodin	C_21_H_22010_	12.83	—	—	—
—	P54	Naringenin-7-O-glucoside	C_21_H_22_O_10_	12.95	—	—	—
—	P55	Notoginsenoside E	C_48_H_82_O_20_	12.96	√	√	√
—	P56	Gymnoside III	C_42_H_58_O_23_	13.22	—	—	—
—	P57	Lobetyolin	C_20_H_28_O_8_	13.24	—	√	—
—	P58	Ginsenoside Re	C_48_H_82_O_18_	13.34	√	—	—
—	P59	Ginsenoside Rg1	C_42_H_72_O_14_	13.40	√	—	√
—	P60	Violanthin	C_27_H_30_O_14_	13.65	—	√	√
—	P61	Militarine	C_34_H_46_O_17_	13.65	√	—	—
—	P62	Ononin/Ononin isomer	C_22_H_22_O_9_	13.73	—	—	—
—	P63	Licorice glycoside B/D1	C_35_H_36_O_15_	13.73	—	—	—
—	P64	Licorice glycoside C2	C_36_H_38_O_16_	13.81	—	—	—
—	P65	N, N′-diferuloylputrescine	C_24_H_28_N_2_O_6_	14.17	—	—	√
—	P66	Licorice glycoside E	C_35_H_35_NO_14_	14.34	—	—	—
—	P67	Pallidiflorin	C_16_H_12_O_4_	14.42	—	√	—
—	P68	Decanedioic acid	C_10_H_18_O_4_	14.45	√	√	√
—	P69	Isoliquiritigenin	C_15_H_12_O_4_	14.46	—	√	√
—	P70	Quercetin	C_15_H_10_O_7_	14.67	—	—	√
—	P71	Licorice saponin A3	C_48_H_72_O_21_	14.69	√	√	—
—	P72	Ginsenoside Rb1	C_54_H_92_O_23_	15.13	√	√	√
—	P73	Licorice saponin G2	C_42_H_62_O_17_	15.24	—	√	—
—	P74	Notoginsenoside R2	C_41_H_70_O_13_	15.31	—	—	√
—	P75	Naringenin	C_15_H_12_O_5_	15.57	—	—	—
—	P76	20S-Ginsenoside Rh1	C_36_H_62_O_9_	15.71	—	—	—
—	P77	Ginsenoside Rh4/Rk3	C_36_H_60_O_8_	15.76	—	—	—
—	P78	Licorice saponin G2 isomer	C_42_H_62_O_17_	15.83	—	—	—
—	P79	Isorhamnetin	C_16_H_12_O_7_	15.95	—	—	—
—	P80	Raho glycyrrhizin	C_48_H_72_O_20_	15.96	—	—	—
—	P81	Betulin	C_30_H_50_O_2_	16.10	—	—	—
—	P82	Ginsenoside Rd	C_51_H_84_O_21_	16.11	—	—	—
—	P83	Yunganoside G1	C_48_H_74_O_21_	16.14	—	—	—
—	P84	Glycyrrhizic acid	C_42_H_62_O_16_	16.31	—	—	—
—	P85	Glycyrrhizic isomer /uralsaponin A/licorice saponin K2/licorice saponin H2	C_42_H_62_O_16_	16.82/17.02	—	—	—
—	P86	Kaikasaponin III	C_48_H_78_O_17_	17.15	—	—	—
—	P87	Uralsaponin C/licorice saponin J2	C_42_H_64_O_16_	17.22	—	—	—
—	P88	Kaikasaponin I	C_42_H_68_O_13_	17.73	—	—	—
—	P89	Paniculatumoside A/paniculatumoside B	C_28_H_40_O_9_	18.00	—	—	—
—	P90	Glyasperin C	C_21_H_24_O_5_	18.09	—	—	—
—	P91	Ginsenoside F2	C_42_H_72_O_13_	18.57	—	—	—
—	P92	Atractylenolide III	C_15_H_20_O_3_	18.62	—	—	—
—	P93	Sophoraisoflavone A/semilicoisoflavone B	C_20_H_16_O_6_	19.91	—	—	—
—	P94	7-[4-(11-Hydroxy-undecyloxy)-phenyl]-7-pyridin-3-yl-hept-6-enoic acid ethyl ester	C_31_H_45_NO_4_	20.82	√	—	√
Total of prototypes					**29**	**27**	**34**

*Metabolites*	*Prototype*	*Biotransformation*	*Formula*	*tR (min)*	*Serum*	*Urine*	*Feces*
M1	P65	Loss of C_14_H_17_NO_3_ + oxidation	C_10_H_11_NO_4_	7.92	—	√	—
M2	P65	Loss of C_14_H_17_NO_3_ + internal hydrolysis	C_10_H_13_NO_4_	10.26	—	√	—
M3	P68	Desaturation	C_10_H_16_O_4_	15.84	—	√	—
M4	P68	Loss of O	C_10_H_18_O_3_	15.32	—	√	—
M5	P68	Loss of O + hydrogenation	C_10_H_20_O_3_	16.94	√	—	—
M6	P65	Loss of C_14_H_18_N_2_O_3_ + ketone formation	C_10_H_8_O_4_	12.16	—	√	—
M7	P27	Loss of O + glucuronidation	C_13_H_14_O_9_	10.48	—	√	—
	P31	Glucuronidation					
M8	P47	Loss of C_27_H_38_O_16_ + ketone formation	C_13_H_16_O_7_	11.22	√	√	—
M9	P47	Loss of C_27_H_38_O_16_ and O	C_13_H_18_O_5_	15.34	—	√	—
M10	P28	Loss of O and C_7_H_4_O_5_ + hydrogenation	C_13_H_18_O_8_	8.74	—	√	—
	P47	Loss of C_27_H_38_O_15_ + oxidation					
M11	P25	Loss of CH_2_	C_13_H_19_NO_4_	6.13	—	√	—
M12	P25	Loss of CH_2_ + sulfate conjugation	C_13_H_19_NO_7_S	9.85	—	√	—
M13	P63	Loss of C_26_H_28_O_13_ + glutamine conjugation	C_14_H_16_N_2_O_4_	13.77	—	√	—
M14	P63	Loss of C_21_H_20_O_9_ and O	C_14_H_16_O_5_	13.34	—	√	—
M15	P63	Loss of C_21_H_20_O_8_	C_14_H_16_O_7_	12.21	—	√	—
	P26	Loss of O and O + hydrogenation					
M16	P63	Loss of C_21_H_20_O_8_ + oxidation	C_14_H_16_O_8_	8.21	—	√	—
M17	P63	Loss of C_21_H_20_O_8_ + oxidation	C_14_H_16_O_8_	9.28	—	√	—
M18	P65	Loss of C_10_H_9_NO_3_ + demethylation to carboxylic acid	C_14_H_17_NO_5_	8.16	—	√	—
M19	P28	Loss of C_7_H_4_O_4_+methylation	C_14_H_18_O_10_	4.85	—	√	—
M20	—P28	Loss of C_7_H_4_O_4_ + methylation	C_14_H_18_O_10_	5.12	—	√	—
M21	—P28	Loss of O and C_7_H_4_O_5_ + methylation	C_14_H_18_O_8_	7.3	—	√	—
	P63	Loss of C_21_H_20_O_8_ + internal hydrolysis					
M22	P28	Loss of O and C_7_H_4_O_5_ + methylation	C_14_H_18_O_8_	11.29	—	√	—
	P63	Loss of C_21_H_20_O_8_ + internal hydrolysis					
M23	P65	Loss of C_10_H_9_NO_3_	C_14_H_19_NO_3_	15.44	—	√	—
M24	P65	Loss of C_10_H_9_NO_3_	C_14_H_19_NO_3_	15.73	—	√	—
M25	P25	Desaturation	C_14_H_19_NO_4_	14.65	—	√	—
M26	P65	Loss of C_10_H_8_O_3_	C_14_H_20_N_2_O_3_	7.03	—	√	—
M27	P65	Loss of C_10_H_8_O_3_ + phosphorylation	C_14_H_21_N_2_O_6_P	5.86	—	√	—
M28	P25	Oxidation	C_14_H_21_NO_5_	2.32	—	—	√
M29	P25	Sulfate conjugation	C_14_H_21_NO_7_S	12.22	—	√	—
M30	P25	Phosphorylation	C_14_H_22_NO_7_P	10.82	—	√	—
M31	P43	Loss of C_12_H_20_O_9_	C_15_H_10_O_7_	14.63	—	√	√
M32	P44	Loss of C_11_H_18_O_9_	C_15_H_12_O_4_	14.47	—	√	√
	P63	Loss of C_20_H_24_O_11_					
M33	P63	Loss of C_20_H_24_O_11_ + oxidation	C_15_H_12_O_5_	15.52	—	√	—
M34	P63	Loss of C_20_H_24_O_12_ + internal hydrolysis	C_15_H_14_O_4_	16.12	—	√	√
M35	P25	Methylation	C_15_H_23_NO_4_	14.47	—	√	—
M36	P47	Loss of C_13_H_16_O_7_ and C_13_H_16_O_6_ + methylation	C_15_H_26_O_9_	13.24	—	√	—
M37	P43	Loss of C_12_H_20_O_10_ and O + methylation	C_16_H_12_O_5_	18.12	—	√	—
M38	P65	Loss of C_14_H_18_N_2_O_3_ + glucose conjugation	C_16_H_20_O_8_	13.53	—	√	—
M39	P63	Loss of C_15_H_10_O_4_ + internal hydrolysis	C_20_H_28_O_12_	8.03	—	√	—
M40	P43	Loss of C_6_H_10_O_4_ + oxidation	C_21_H_20_O_13_	9.06	—	√	—
M41	P28	Loss of O and O + methylation	C_21_H_22_O_12_	8.03	—	√	—
M42	P63	Loss of C_9_H_6_O_2_ + glucuronidation	C_32_H_38_O_19_	9.94	—	√	—
M43	P59	Loss of O	C_42_H_72_O_13_	15.11	√	√	√
M44	P27	Loss of O and O	C_7_H_6_O_2_	12.52	—	√	—
	P31	Loss of O					
M45	P23	Loss of O and O	C_7_H_6_O_3_	13.51	√	√	—
	P27	Loss of O					
	P28	Loss of O and C_13_H_14_O_10_					
	P47	Loss of C_27_H_38_O_15_ and C_6_H_10_O_6_ + demethylation to carboxylic acid					
M46	P23	Loss of O	C_7_H_6_O_4_	9.12	—	√	—
	P28	Loss of C_13_H_14_O_10_					
	P31	Oxidation					
M47	P27	Oxidation	C_7_H_6_O_5_	4.16	—	√	√
	P28	Loss of C_13_H_14_O_9_					
M48	P27	Loss of O + sulfate conjugation	C_7_H_6_O_6_S	6.83	√	√	—
	P31	Sulfate conjugation					
M49	P23	Loss of O + sulfate conjugation	C_7_H_6_O_7_S	6.76	√	√	—
	P36	Loss of C_20_H_16_O_14_ + sulfate conjugation					
	P27	Sulfate conjugation					
M50	P27	Loss of O and O + hydrogenation	C_7_H_8_O_2_	8.01	—	√	√
	P31	Loss of O + hydrogenation					
	P47	Loss of C_27_H_38_O_15_ and C_6_H_10_O_5_					
M51	P27	Loss of O and O + methylation	C_8_H_8_O_2_	11.44	—	√	—
	P31	Loss of O + methylation					
M52	P36	Loss of C_20_H_16_O_14_ and O + methylation	C_8_H_8_O_3_	13.29	—	√	—
	P26	Loss of C_7_H_8_O_5_ and O + methylation					
	P23	Loss of O and O + methylation					
	P27	Loss of O + methylation					
	P31	Methylation					
M53	P23	Loss of O + methylation	C_8_H_8_O_4_	12.58	√	√	—
	P27	Methylation					
	P36	Loss of C_20_H_16_O_14_ + methylation					
	P28	Loss of C_13_H_14_O_10_ + methylation					
M54	P23	Methylation	C_8_H_8_O_5_	8.53	√	√	√
	P36	Loss of C_20_H_16_O_13_ + methylation					
	P26	Loss of C_7_H_8_O_4_ + methylation					
	P28	Loss of C_13_H_14_O_9_ + methylation					
M55	P63	Loss of C_26_H_28_O_13_ + internal hydrolysis	C_9_H_10_O_3_	9	√	√	—
M56	P63	Loss of C_26_H_28_O_12_	C_9_H_8_O_3_	11.56	√	√	√
	P65	Loss of CH_2_ and C_14_H_18_N_2_O_3_					
M57	P63	Loss of C_26_H_28_O_12_ + oxidation	C_9_H_8_O_4_	14.04	—	—	√
M58	P63	Loss of C_26_H_28_O_12_ + oxidation	C_9_H_8_O_4_	9.04	—	√	—
M59	P50	Glucuronidation	C_34_H_40_O_22_	10.68	—	√	—
M60	P37	Ketone formation	C_33_H_38_O_22_	9.37	—	√	—
	P43	Glucuronidation					
M61	P47	Loss of C_13_H_16_O_7_ and O + phosphorylation	C_27_H_41_O_17_P	5.05	—	√	—
M62	P50	Demethylation to carboxylic acid	C_28_H_30_O_18_	10.57	—	√	—
M63	P59	Loss of C_6_H_10_O_6_	C_36_H_62_O_8_	21.05	—	—	√
	P58	Loss of C_12_H_20_O_10_					
M64	P37	Loss of O and C_6_H_10_O_6_ + hydrogenation	C_27_H_32_O_14_	13.05	—	—	√
	P43	Loss of O and O + hydrogenation					
M65	P50	Loss of C_6_H_10_O_5_ + demethylation to carboxylic acid	C_22_H_20_O_13_	10.6	—	√	—
M66	P84	Loss of C_12_H_16_O_12_ + oxidation	C_30_H_46_O_5_	19.88	—	—	√
	P71	Loss of C_12_H_16_O_12_ and C_6_H_10_O_5_ + oxidation					
M67	P84	Loss of C_12_H_16_O_12_ + oxidation	C_30_H_46_O_5_	19.47	√	—	√
	P71	Loss of C_12_H_16_O_12_ and C_6_H_10_O_5_ + oxidation					
M68	P50	Loss of C_6_H_10_O_4_	C_22_H_22_O_12_	13.16	√	√	—
	P37	Loss of C_12_H_20_O_9_ + methylation					
	P43	Loss of C_6_H_10_O_4_ + methylation					
M69	P37	Loss of C_12_H_20_O_10_ + demethylation to carboxylic acid	C_21_H_18_O_13_	13.13	√	√	—
	P43	Loss of C_6_H_10_O_5_ + demethylation to carboxylic acid					
M70	P50	Loss of C_6_H_10_O_4_ and O + ketone formation	C_22_H_20_O_12_	12.83	—	√	—
M71	P50	Loss of C_6_H_10_O_4_ and O + ketone formation	C_22_H_20_O_12_	12.51	—	√	—
M72	P84	Loss of C_12_H_16_O_12_	C_30_H_46_O_4_	22.29	√	—	—
	P71	Loss of C_12_H_16_O_12_ and C_6_H_10_O_5_					
M73	P84	Loss of C_12_H_16_O_13_ + ketone formation	C_30_H_44_O_4_	19.47	√	√	√
M74	P50	Loss of C_6_H_10_O_5_	C_22_H_22_O_11_	15.04	—	√	—
	P37	Loss of C_12_H_20_O_10_ + methylation					
	P43	Loss of C_6_H_10_O_5_ + methylation					
M75	P50	Loss of C_6_H_10_O_5_ + demethylation and methylene to ketone	C_21_H_18_O_12_	13.1	—	√	—
	P37	Loss of C_12_H_20_O_10_ + ketone formation					
	P43	Loss of C_6_H_10_O_5_ + ketone formation					
M76	P50	Loss of C_6_H_10_O_5_ + demethylation and methylene to ketone	C_21_H_18_O_12_	12.79	—	√	—
	P37	Loss of C_12_H_20_O_10_ + ketone formation					
	P43	Loss of C_6_H_10_O_5_ + ketone formation					
M77	P50	Loss of C_6_H_10_O_4_ and CH_2_O	C_21_H_20_O_11_	13.08	√	√	—
	P37	Loss of C_12_H_20_O_10_					
	P43	Loss of C_6_H_10_O_5_					
M78	P50	Loss of C_6_H_10_O_4_ and CH_2_O	C_21_H_20_O_11_	12.95	√	√	—
	P37	Loss of C_12_H_20_O_10_					
	P43	Loss of C_6_H_10_O_5_					
M79	P44	Loss of C_5_H_8_O_4_ + oxidation	C_21_H_22_O_10_	13.79	—	√	—
	P37	Loss of O and C_12_H_20_O_10_ + hydrogenation					
	P43	Loss of C_6_H_10_O_5_ and O + hydrogenation					
M80	P44	Loss of C_5_H_8_O_5_ + demethylation to carboxylic acid	C_21_H_20_O_10_	11.83	√	√	—
	P50	Loss of C_6_H_10_O_5_ and CH_2_O					
	P37	Loss of O and C_12_H_20_O_10_					
	P43	Loss of C_6_H_10_O_5_ and O					
M81	P44	Loss of C_5_H_8_O_5_+ketone formation	C_21_H_20_O_9_	10.5	—	√	—
M82	P44	Loss of C_5_H_8_O_5_+hydrogenation	C_21_H_24_O_8_	13.18	—	√	—
M83	P6	Loss of H–_2_O + methylation	C_13_H_26_O_10_	3.74	—	√	—
M84	P47	Loss of C_13_H_16_O_7_ and C_13_H_16_O_6_ + demethylation and methylene to ketone	C_13_H_20_O_10_	7.5	—	√	—
M85	P58	Loss of C_36_H_60_O_9_ + methylation	C_13_H_24_O_9_	6.42	—	√	—
	P50	Loss of C_16_H_10_O_7_ + methylation					
	P37	Loss of C_21_H_18_O_12_ + methylation					
	P7	Loss of C_6_H_10_O_6_ and O + methylation					
	P43	Loss of C_15_H_8_O_7_ + methylation					
	P6	Loss of O and O + methylation					
	P8	Loss of C_6_H_10_O_6_ and C_6_H_10_O_6_ + methylation					
M86	P47	Loss of C_27_H_38_O_15_ + methylation	C_14_H_20_O_7_	11.44	—	√	—
	P74	Loss of C_21_H_28_O_10_ + methylation					
M87	P50	Loss of C_12_H_20_O_9_ and CH_2_O	C_15_H_10_O_6_	13.08	—	√	—
	P37	Loss of C_18_H_30_O_15_					
	P43	Loss of C_12_H_20_O_10_					
M88	P47	Loss of C_27_H_38_O_16_	C_13_H_18_O_6_	10.65	—	√	√
	P74	Loss of C_21_H_28_O_11_					
M89	P47	Loss of C_27_H_38_O_16_ + loss of hydroxymethylene	C_12_H_16_O_5_	11.17	—	√	—
	P74	Loss of C_21_H_28_O_11_ + loss of hydroxymethylene					
M90	P44	Loss of C_11_H_18_O_10_	C_15_H_12_O_3_	10.5	—	√	—
M91	P12	Loss of O + loss of hydroxymethylene	C_9_H_11_N_5_O_2_	1.38	—	—	√
M92	P74	Loss of C_13_H_16_O_6_ and C_13_H_16_O_6_	C_8_H_14_O_5_	11.2	—	√	—
M93	P36	Loss of C_20_H_16_O_13_ + decarboxylation	C_6_H_6_O_3_	6	—	√	—
M94	P26	Loss of C_7_H_4_O_5_	C_7_H_10_O_4_	14.59	—	√	—
M95	P36	Loss of C_20_H_16_O_14_ + taurine conjugation	C_9_H_11_NO_6_S	2.81	√	—	—
M96	P36	Loss of C_14_H_6_O_10_ + demethylation and methylene to ketone	C_12_H_12_O_9_	4.71	—	√	—
M97	P23	Loss of O and O + glucose conjugation	C_13_H_16_O_8_	4.11	—	√	—
	P36	Loss of C_14_H_6_O_10_					
	P26	Loss of C_7_H_8_O_5_ and O + glucose conjugation					
	P31	Glucose conjugation					
	P47	Loss of C_27_H_38_O_16_ + demethylation to carboxylic acid					
M98	P36	Loss of C_14_H_6_O_10_ + hydrogenation	C_13_H_18_O_8_	8.74	—	√	—
M99	P36	Loss of C_13_H_12_O_10_	C_14_H_10_O_8_	13.63	—	√	—
M100	P23	Loss of O + glucose conjugation	C_13_H_16_O_9_	6.81	√	√	—
	P27	Glucose conjugation					
	P28	Loss of C_7_H_4_O_5_					
	P36	Loss of C_14_H_6_O_9_					
	P26	Loss of C_7_H_8_O_5_ + glucose conjugation					
M101	P23	Loss of O + glucose conjugation	C_13_H_16_O_9_	6.57	√	√	—
	P27	Glucose conjugation					
	P28	Loss of C_7_H_4_O_5_					
	P36	Loss of C_14_H_6_O_9_					
	P26	Loss of C_7_H_8_O_5_ + glucose conjugation					
M102	P23	Loss of O + glucuronidation	C_13_H_14_O_10_	5.69	—	√	—
	P26	Loss of C_7_H_8_O_5_ + glucuronidation					
	P27	Glucuronidation					
	P28	Loss of C_7_H_4_O_5_ + ketone formation					
M103	P36	Loss of C_14_H_6_O_9_ + methylation	C_14_H_18_O_9_	3.63	—	√	—
M104	P13	Loss of H–_2_O + hydrogenation	C_14_H_16_O_10_	7.96	—	√	—
M105	P36	Loss of C_13_H_12_O_8_ + methylation	C_15_H_12_O_10_	11.93	—	√	—
M106	P36	Loss of C_13_H_12_O_8_ + methylation	C_15_H_12_O_10_	10.7	—	√	—
M107	P36	Loss of C_13_H_12_O_9_ + glutamine conjugation	C_19_H_18_N_2_O_11_	14.07	—	—	√
**Total of metabolites**					**22**	**96**	**18**

## Data Availability

The data used to support the findings of this study are included within the article.

## References

[1] Cheung F. (2011). TCM: made in China. *Nature*.

[2] Liu Z., Guo F., Wang Y. (2016). BATMAN-TCM: a bioinformatics analysis tool for molecular mechANism of traditional Chinese medicine. *Scientific Reports*.

[3] Han Y., Sun H., Zhang A., Yan G., Wang X.-J. (2020). Chinmedomics, a new strategy for evaluating the therapeutic efficacy of herbal medicines. *Pharmacology & Therapeutics*.

[4] Xin G.-Z., Qi L.-W., Shi Z.-Q. (2011). Strategies for integral metabolism profile of multiple compounds in herbal medicines: pharmacokinetics, metabolites characterization and metabolic interactions. *Current Drug Metabolism*.

[5] Feuerstein J. D., Moss A. C., Farraye F. A. (2019). Ulcerative colitis. *Mayo Clinic Proceedings*.

[6] Jing Y., Li A., Liu Z. (2018). Absorption of Codonopsis pilosula saponins by coexisting polysaccharides alleviates gut microbial dysbiosis with dextran sulfate sodium-induced colitis in model mice. *BioMed Research International*.

[7] Feng W., Liu J., Tan Y., Ao H., Wang J., Peng C. (2020). Polysaccharides from Atractylodes macrocephala Koidz. Ameliorate ulcerative colitis via extensive modification of gut microbiota and host metabolism. *Food research international (Ottawa, Ont.)*.

[8] Habtemariam S., Belai A. (2018). Natural therapies of the inflammatory bowel disease: the case of rutin and its aglycone, quercetin. *Mini Reviews in Medicinal Chemistry*.

[9] Chen Y., Yu R., Jiang L. (2019). A comprehensive and rapid quality evaluation method of traditional Chinese medicine decoction by integrating UPLC-QTOF-MS and UFLC-QQQ-MS and its application. *Molecules*.

[10] Wang F., Huang S., Chen Q. (2020). Chemical characterisation and quantification of the major constituents in the Chinese herbal formula Jian‐Pi‐Yi‐Shen pill by UPLC‐Q‐TOF‐MS/MS and HPLC‐QQQ‐MS/MS. *Phytochemical Analysis*.

[11] Liu M.-H., Tong X., Wang J.-X., Zou W., Cao H., Su W.-W. (2013). Rapid separation and identification of multiple constituents in traditional Chinese medicine formula Shenqi Fuzheng Injection by ultra-fast liquid chromatography combined with quadrupole-time-of-flight mass spectrometry. *Journal of Pharmaceutical and Biomedical Analysis*.

[12] Tao W.-W., Duan J.-A., Yang N.-Y. (2012). Determination of nucleosides and nucleobases in the pollen of Typha angustifolia by UPLC-PDA-MS. *Phytochemical Analysis*.

[13] Li J., Wang R.-F., Zhou Y. (2019). Dammarane-type triterpene oligoglycosides from the leaves and stems of Panax notoginseng and their antiinflammatory activities. *Journal of Ginseng Research*.

[14] Xie Q., Yuan H., Liu Y. (2019). Simultaneous determination of 19 bioactive constituents in QishenYiqi dropping pills by ultra-performance liquid chromatography coupled with triple quadrupole mass spectrometry. *Journal of AOAC International*.

[15] Gu C.-Z., Lv J.-J., Zhang X.-X. (2015). Triterpenoids with promoting effects on the differentiation of PC12 cells from the steamed roots of Panax notoginseng. *Journal of Natural Products*.

[16] Xu D., Pan Y., Chen J. (2019). Chemical constituents, pharmacologic properties, and clinical applications of Bletilla striata. *Frontiers in Pharmacology*.

[17] Zhao Y., Niu J.-J., Cheng X.-C. (2018). Chemical constituents from Bletilla striata and their NO production suppression in RAW 264.7 macrophage cells. *Journal of Asian Natural Products Research*.

[18] Lo Y.-H., Lin R.-D., Lin Y.-P., Liu Y.-L., Lee M.-H. (2009). Active constituents from Sophora japonica exhibiting cellular tyrosinase inhibition in human epidermal melanocytes. *Journal of Ethnopharmacology*.

[19] Zhang Y., Qu L., Liu L. (2015). New maltol glycosides from flos sophorae. *Journal of Natural Medicines*.

[20] Zhang W., Jiang H., Yang J. (2019). Safety assessment and antioxidant evaluation of betulin by LC-MS combined with free radical assays. *Analytical Biochemistry*.

[21] Liu R., Cai Z., Xu B. (2017). Characterization and quantification of flavonoids and saponins in adzuki bean (Vigna angularis L.) by HPLC-DAD-ESI-MSn analysis. *Chemistry Central Journal*.

[22] Kite G. C., Stoneham C. A., Veitch N. C. (2007). Flavonol tetraglycosides and other constituents from leaves of Styphnolobium japonicum (Leguminosae) and related taxa. *Phytochemistry*.

[23] Wang L., Chen C., Su A., Zhang Y., Yuan J., Ju X. (2016). Structural characterization of phenolic compounds and antioxidant activity of the phenolic-rich fraction from defatted adlay (Coix lachryma-jobi L . var. ma-yuen Stapf) seed meal. *Food Chemistry*.

[24] Chen Y., Yu H., Wu H. (2015). Characterization and quantification by LC-MS/MS of the chemical components of the heating products of the flavonoids extract in pollen Typhae for transformation rule exploration. *Molecules*.

[25] Xu M., Jin Z., Ohm J.-B., Schwarz P., Rao J., Chen B. (2019). Effect of germination time on antioxidative activity and composition of yellow pea soluble free and polar soluble bound phenolic compounds. *Food & Function*.

[26] Jin X., Lu Y., Chen S., Chen D. (2020). UPLC-MS identification and anticomplement activity of the metabolites of Sophora tonkinensis flavonoids treated with human intestinal bacteria. *Journal of Pharmaceutical and Biomedical Analysis*.

[27] Cedeño H., Espinosa S., Andrade J. M., Cartuche L., Malagón O. (2019). Novel flavonoid glycosides of quercetin from leaves and flowers of gaiadendron punctatum G.don. (violeta de Campo), used by the saraguro community in southern Ecuador, inhibit *α*-glucosidase enzyme. *Molecules*.

[28] Yang W.-Y., Won T. H., Ahn C.-H. (2015). Streptococcus mutans sortase A inhibitory metabolites from the flowers of Sophora japonica. *Bioorganic & Medicinal Chemistry Letters*.

[29] Ryu J., Kim J. S., Kang S. S. (2003). Cerebrosides from longan arillus. *Archives of Pharmacal Research*.

[30] Wang G., Cui Q., Yin L.-J. (2019). Efficient extraction of flavonoids from Flos Sophorae Immaturus by tailored and sustainable deep eutectic solvent as green extraction media. *Journal of Pharmaceutical and Biomedical Analysis*.

[31] Yin Q., Wang P., Zhang A., Sun H., Wu X., Wang X. (2013). Ultra-performance LC-ESI/quadrupole-TOF MS for rapid analysis of chemical constituents of Shaoyao-Gancao decoction. *Journal of Separation Science*.

[32] Schmid C., Dawid C., Peters V., Hofmann T. (2018). Saponins from European licorice roots (Glycyrrhiza glabra). *Journal of Natural Products*.

[33] Farag M. A., Porzel A., Wessjohann L. A. (2012). Comparative metabolite profiling and fingerprinting of medicinal licorice roots using a multiplex approach of GC-MS, LC-MS and 1D NMR techniques. *Phytochemistry*.

[34] Xu T., Yang M., Li Y. (2013). An integrated exact mass spectrometric strategy for comprehensive and rapid characterization of phenolic compounds in licorice. *Rapid Communications in Mass Spectrometry*.

[35] Shan L., Yang N., Zhao Y., Sheng X., Yang S., Li Y. (2018). A rapid classification and identification method applied to the analysis of glycosides in Bupleuri radix and liquorice by ultra high performance liquid chromatography coupled with quadrupole time-of-flight mass spectrometry. *Journal of Separation Science*.

[36] Song W., Qiao X., Chen K. (2017). Biosynthesis-based quantitative analysis of 151 secondary metabolites of licorice to differentiate medicinal Glycyrrhiza species and their hybrids. *Analytical Chemistry*.

[37] Li Z., Liu T., Liao J., Ai N., Fan X., Cheng Y. (2017). Deciphering chemical interactions between Glycyrrhizae Radix and Coptidis Rhizoma by liquid chromatography with transformed multiple reaction monitoring mass spectrometry. *Journal of Separation Science*.

[38] Fang S., Qu Q., Zheng Y. (2016). Structural characterization and identification of flavonoid aglycones in threeGlycyrrhizaspecies by liquid chromatography with photodiode array detection and quadrupole time-of-flight mass spectrometry. *Journal of Separation Science*.

[39] Li S.-L., Tan H., Shen Y.-M., Kawazoe K., Hao X.-J. (2004). A pair of new C-21 steroidal glycoside epimers from the roots of Cynanchum paniculatum. *Journal of Natural Products*.

[40] Wang X., Chen X., Li J. (2020). Thrombin-based discovery strategy of bioactive-chemical quality marker combination for pollen of Typha orientalis by metabolomics coupled with chemometrics. *Phytomedicine*.

[41] Ding M., Jiang Y., Yu X. (2018). Screening of combinatorial quality markers for natural products by metabolomics coupled with chemometrics. A case study on pollen Typhae. *Frontiers in Pharmacology*.

[42] Lee D. Y., Yang H., Kim H. W., Sung S. H. (2017). New polyhydroxytriterpenoid derivatives from fruits of Terminalia chebula Retz. and their *α*-glucosidase and *α*-amylase inhibitory activity. *Bioorganic & Medicinal Chemistry Letters*.

[43] Sarabhai S., Sharma P., Capalash N. (2013). Ellagic acid derivatives from Terminalia chebula Retz. downregulate the expression of quorum sensing genes to attenuate *Pseudomonas aeruginosa* PAO1 virulence. *Plos One*.

[44] Pfundstein B., El Desouky S. K., Hull W. E., Haubner R., Erben G., Owen R. W. (2010). Polyphenolic compounds in the fruits of Egyptian medicinal plants (Terminalia bellerica, Terminalia chebula and Terminalia horrida): characterization, quantitation and determination of antioxidant capacities. *Phytochemistry*.

[45] Fahmy N. M., Al-Sayed E., Abdel-Daim M. M., Karonen M., Singab A. N. (2016). Protective effect ofTerminalia muelleriagainst carbon tetrachloride-induced hepato and nephro-toxicity in mice and characterization of its bioactive constituents. *Pharmaceutical Biology*.

[46] Li K., Han X., Li R. (2019). Composition, antivirulence activity, and active property distribution of the fruit ofTerminalia chebulaRetz. *Journal of Food Science*.

[47] Pellati F., Bruni R., Righi D. (2013). Metabolite profiling of polyphenols in a Terminalia chebula Retzius ayurvedic decoction and evaluation of its chemopreventive activity. *Journal of Ethnopharmacology*.

[48] Lee D. Y., Kim H. W., Yang H., Sung S. H. (2017). Hydrolyzable tannins from the fruits of Terminalia chebula Retz and their *α*-glucosidase inhibitory activities. *Phytochemistry*.

[49] Sun X., Cui X.-B., Wen H.-M. (2017). Influence of sulfur fumigation on the chemical profiles of Atractylodes macrocephala Koidz. evaluated by UFLC-QTOF-MS combined with multivariate statistical analysis. *Journal of Pharmaceutical and Biomedical Analysis*.

[50] Lin L., Yang Q., Zhao K., Zhao M. (2018). Identification of the free phenolic profile of Adlay bran by UPLC-TOF-MS/MS and inhibitory mechanisms of phenolic acids against xanthine oxidase. *Food Chemistry*.

[51] Zhang A., Zou D., Yan G., Tan Y., Sun H., Wang X. (2014). Identification and characterization of the chemical constituents of Simiao Wan by ultra high performance liquid chromatography with mass spectrometry coupled to an automated multiple data processing method. *Journal of Separation Science*.

[52] Lu J.-J., Hu X.-W., Li P., Chen J. (2017). Global identification of chemical constituents and rat metabolites of Si-Miao-Wan by liquid chromatography-electrospray ionization/quadrupole time-of-flight mass spectrometry. *Chinese Journal of Natural Medicines*.

[53] Kim S. Y., Choi C. W., Hong S. S., Shin H., Oh J. S. (2016). A New Neolignan from Coix lachryma-jobi var. mayuen. *Natural product communications*.

[54] Andrade M. E. R., Santos R. D. G. C. D., Soares A. D. N. (2016). Pretreatment and treatment WithL-arginine attenuate weight loss and bacterial translocation in dextran sulfate sodium colitis. *Journal of Parenteral and Enteral Nutrition*.

[55] Ren W., Yin J., Wu M. (2014). Serum amino acids profile and the beneficial effects of L-arginine or L-glutamine supplementation in dextran sulfate sodium colitis. *Plos One*.

[56] Coburn L. A., Gong X., Singh K. (2012). L-arginine supplementation improves responses to injury and inflammation in dextran sulfate sodium colitis. *Plos One*.

[57] Omidi-Ardali H., Lorigooini Z., Soltani A., Balali-Dehkordi S., Amini-Khoei H. (2019). Inflammatory responses bridge comorbid cardiac disorder in experimental model of IBD induced by DSS: protective effect of the trigonelline. *Inflammopharmacology*.

[58] Jin J., Zhong Y., Long J. (2021). Ginsenoside Rg1 relieves experimental colitis by regulating balanced differentiation of Tfh/Treg cells. *International Immunopharmacology*.

[59] Choi Y. H., Bae J.-K., Chae H.-S. (2016). Isoliquiritigenin ameliorates dextran sulfate sodium-induced colitis through the inhibition of MAPK pathway. *International Immunopharmacology*.

[60] Yang X.-L., Guo T.-K., Wang Y.-H., Gao M.-T., Qin H., Wu Y.-J. (2012). Therapeutic effect of ginsenoside Rd in rats with TNBS-induced recurrent ulcerative colitis. *Archives of Pharmacal Research*.

[61] Yang N., Liang G., Lin J. (2020). Ginsenoside Rd therapy improves histological and functional recovery in a rat model of inflammatory bowel disease. *Phytotherapy Research*.

[62] Wang X.-r., Hao H.-g., Chu L. (2017). Glycyrrhizin inhibits LPS-induced inflammatory mediator production in endometrial epithelial cells. *Microbial Pathogenesis*.

[63] Ali H., Weigmann B., Collnot E.-M., Khan S. A., Windbergs M., Lehr C.-M. (2016). Budesonide loaded PLGA nanoparticles for targeting the inflamed intestinal mucosa-pharmaceutical characterization and fluorescence imaging. *Pharmaceutical Research*.

[64] Zeeshan M., Ali H., Khan S., Mukhtar M., Khan M. I., Arshad M. (2019). Glycyrrhizic acid-loaded pH-sensitive poly-(lactic-co-glycolic acid) nanoparticles for the amelioration of inflammatory bowel disease. *Nanomedicine*.

[65] Mao X., Sun R., Wang Q. (2021). l-Isoleucine administration alleviates DSS-induced colitis by regulating TLR4/MyD88/NF-kappaB pathway in rats. *Frontiers in Immunology*.

[66] Jeengar M. K., Thummuri D., Magnusson M., Naidu V. G. M., Uppugunduri S. (2017). Uridine ameliorates dextran sulfate sodium (DSS)-Induced colitis in mice. *Scientific Reports*.

[67] Zizzo M. G., Caldara G., Bellanca A., Nuzzo D., Di Carlo M., Serio R. (2019). Preventive effects of guanosine on intestinal inflammation in 2, 4-dinitrobenzene sulfonic acid (DNBS)-induced colitis in rats. *Inflammopharmacology*.

[68] Zhu L., Gu P., Shen H. (2019). Gallic acid improved inflammation via NF-*κ*B pathway in TNBS-induced ulcerative colitis. *International Immunopharmacology*.

[69] Pandurangan A. K., Mohebali N., MohdEsa N., Looi C. Y., Ismail S., Saadatdoust Z. (2015). Gallic acid suppresses inflammation in dextran sodium sulfate-induced colitis in mice: possible mechanisms. *International Immunopharmacology*.

[70] Mascaraque C., Aranda C., Ocón B. (2014). Rutin has intestinal antiinflammatory effects in the CD4+ CD62L+ T cell transfer model of colitis. *Pharmacological Research*.

[71] Kim H., Kong H., Choi B. (2005). Metabolic and pharmacological properties of rutin, a dietary quercetin glycoside, for treatment of inflammatory bowel disease. *Pharmaceutical Research*.

[72] Kim S.-J., Kim M.-C., Um J.-Y., Hong S.-H. (2010). The beneficial effect of vanillic acid on ulcerative colitis. *Molecules*.

[73] Bian Y., Liu P., Zhong J. (2018). Quercetin attenuates adhesion molecule expression in intestinal microvascular endothelial cells by modulating multiple pathways. *Digestive Diseases and Sciences*.

[74] Dong Y., Lei J., Zhang B. (2020). Dietary quercetin alleviated DSS-induced colitis in mice through several possible pathways by transcriptome analysis. *Current Pharmaceutical Biotechnology*.

[75] Zhang J., Cao L., Wang H. (2015). Ginsenosides regulate PXR/NF-*κ*B signaling and attenuate dextran sulfate sodium-induced colitis. *Drug Metabolism and Disposition*.

[76] El-Sherbiny M., Eisa N. H., Abo El-Magd N. F., Elsherbiny N. M., Said E., Khodir A. E. (2021). Anti-inflammatory/anti-apoptotic impact of betulin attenuates experimentally induced ulcerative colitis: an insight into TLR4/NF-kB/caspase signalling modulation. *Environmental Toxicology and Pharmacology*.

[77] He Q., He L., Zhang F. (2020). Stachyose modulates gut microbiota and alleviates dextran sulfate sodium-induced acute colitis in mice. *Saudi Journal of Gastroenterology*.

[78] Cheng C., Zhang W., Zhang C. (2021). Hyperoside ameliorates DSS-induced colitis through MKRN1-mediated regulation of PPAR*γ* signaling and Th17/treg balance. *Journal of Agricultural and Food Chemistry*.

